# Mechanical Tuning of Singlet–Triplet Resonance for Efficient Triplet–Triplet Annihilation Upconversion

**DOI:** 10.1021/acs.jctc.6c01488

**Published:** 2026-09-25

**Authors:** Edgard Miranda-Sáenz, Pedro B. Coto, Luis Manuel Frutos

**Affiliations:** † Nanomaterials and Nanotechnology Research Center (CINN) Spanish National Research Council (CSIC) and Donostia International Physics Center (DIPC), Avenida de la Vega 4−6, El Entrego 33940, Spain; ‡ Universidad de Alcalá, Departamento de Química Analítica, Química Física e Ingeniería Química, Grupo de Reactividad y Estructura Molecular (RESMOL), Alcalá de Henares, 28805 Madrid, Spain; § Universidad de Alcalá, Instituto de Investigación Química ‘Andrés M. del Río’’ (IQAR), Alcalá de Henares, 28805 Madrid, Spain

## Abstract

Triplet–triplet annihilation upconversion depends on a near-resonant energetic balance between the relevant singlet and triplet excited states. Here we introduce a computational framework for mechanically tuning this balance by applying an external force that permits to locate the molecular geometry reaching the singlet–triplet resonance condition, i.e., *E*_*S*_1__ – *E*_*S*_0__ = 2­(*E*_*T*_1__ – *E*_*S*_0__). Our approach formulates this problem as a constrained optimization in which the force magnitude is minimized while enforcing the target energetic resonance condition. Using a local quadratic model for the triplet-state potential energy surface together with a linearized description of the relevant energy gaps, we derive a minimum-force update procedure to obtain the optimal displacement and the associated external force. The resulting algorithm provides the mechanically controlled geometry and the optimal force vector required to reach it. The method offers a practical computational route to identify mechanically accessible structures with improved upconversion characteristics, paving the way for the design of mechanoresponsive chromophores and materials with stress-tunable optical behavior.

## Graphical abstract

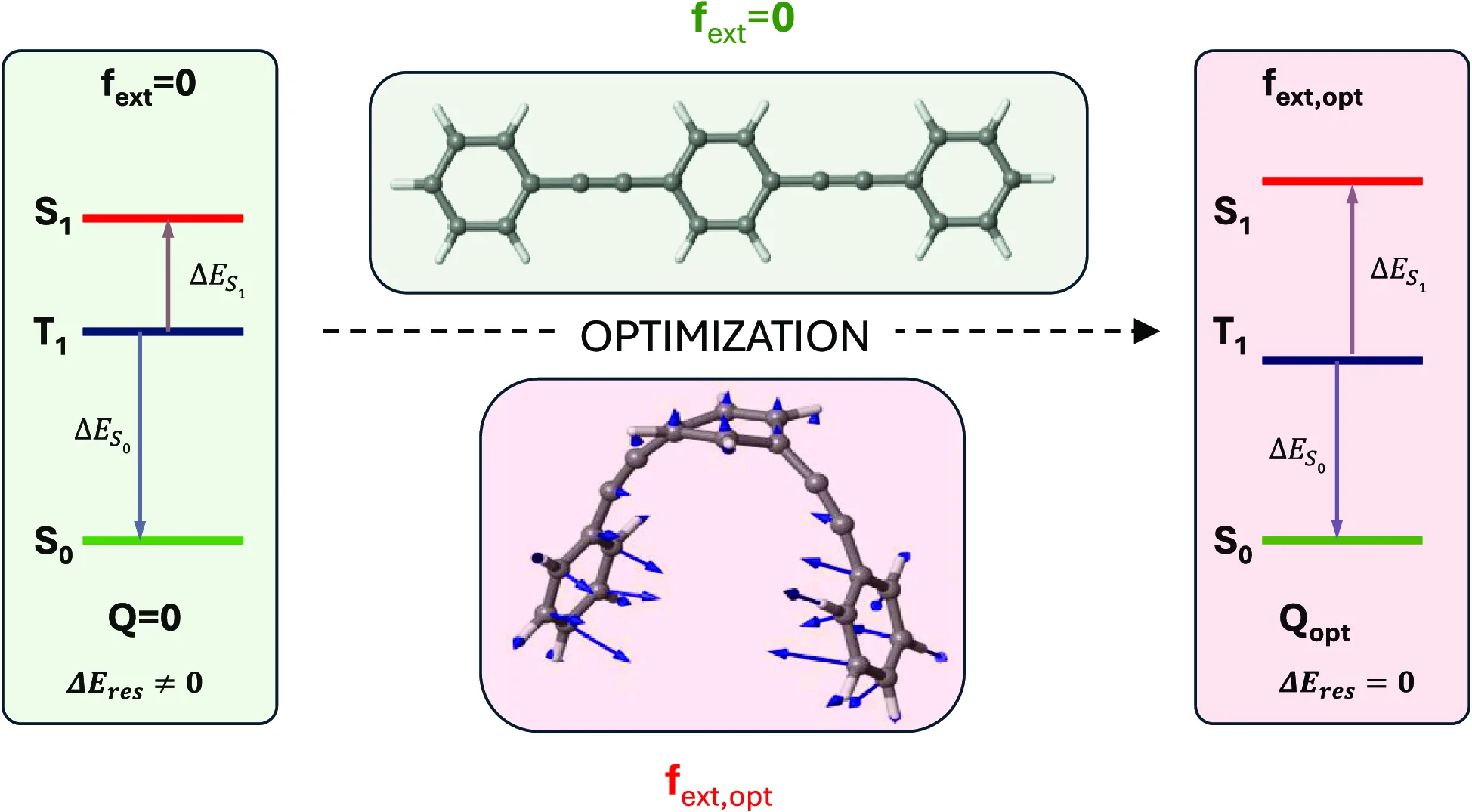

## Introduction

Mechanochemistry has emerged as a flexible strategy for promoting and controlling chemical reactivity through the direct action of mechanical forces on chemical systems.^1^ At the molecular level, covalent mechanochemistry makes use of atomically directed forces to control potential energy surfaces (PES), alter energy barriers, and bias reaction pathways in a selective manner.^2^ In polymer systems, force can be transmitted through macromolecular backbones to activate mechanophores and generate changes in bonding,^3^ color,^4^ luminescence,^5^ or reactivity.^6^ On the other hand, bulk mechanochemistry, particularly ball milling, has become a powerful platform for synthesis and materials transformation,^7^ where repeated impact, friction, and compression events induce chemical change under far-from-equilibrium conditions and often with reduced solvent requirements.^8^ Therefore, these developments establish that mechanical forces are not just an energy input, but a tool for molecular and materials design.

The extension of mechanochemical concepts to electronically excited states is also possible because mechanical forces can, in principle, modulate electronic states energy,^9^ photochemical reactivity^10^ and photophysical behavior.^11^ Force-induced distortions may tune excitation and emission energies,^12^ alter excited-state relaxation channels, and modify the accessibility to reactive regions of the excited-states.^13^ Previous work has shown that mechanical stress can regulate photochemical outcomes^14^ and even change the topology of conical intersections,^15^ opening the possibility of steering light-driven processes through directed force.^16^ Nevertheless, mechano-photochemistry, i.e., mechanical control of photochemical processes, remains at an early stage, and a clear understanding of how mechanical forces affect excited-state spectra, nonadiabatic pathways, and radiationless decay channels is still developing.^10^

From a theory and simulation perspective, the available theoretical models and computational tools are relatively limited. For covalent mechanochemistry under atomically controlled forces, several quantum-chemical approaches have been developed to analyze force induced reactivity and to construct force-modified PESs.^2^ In contrast, computational descriptions of bulk mechanochemistry, including ball-milling environments, are less developed because they must connect electronic structure with complex modes of mechanical-energy transfer in heterogeneous condensed-phase systems.^17^ Here, molecular mechanics or hybrid quantum mechanics-molecular mechanics methods have been employed in describing the dynamics of complex systems under the effect of macroscopic forces,^18^ and simple theoretical models have been proposed to determine the mechanical effect of ball-milling impacts on chemical reactivity.^19^

This limitation becomes even more pronounced for excited states, where reliable modeling often requires the simultaneous treatment of multiple PESs, nonadiabatic couplings, and ultrafast relaxation processes.^20^ Thus, although the control of photoinduced processes using mechanical forces (as is the case of mechano-photocatalysis) is attracting increasing attention, the understanding of how these forces influence excited-state landscape, spectra, and nonadiabatic reaction pathways remains very limited from the fundamental point of view, thus hindering the use of mechano-photochemistry to control photoinduced processes.^21^

In this context, triplet–triplet annihilation upconversion (TTA-UC), is a relevant photoinduced process that enables the conversion of two low-energy photons into one higher-energy photon under relatively low excitation intensities.^22^ These characteristics make TTA-UC relevant for important applications such as solar-energy harvesting,^23^ photocatalysis,^24^ sensing,^25^ bioimaging,^26^ and optoelectronic technologies.^27^ Because TTA-UC efficiency depends strongly on the energy relationship between the relevant singlet and triplet excited states, even small structural perturbations can have a major impact on upconversion performance. Mechanical control therefore offers a direct way to regulate TTA-UC through targeted modulation of excited-state energetics.^12^

In this work, we present the development and implementation a computational model designed to predict the optimal external forces required to control TTA-UC. Specifically, the algorithm finds the minimum force magnitude that has to be applied to a molecular system to reach the desired singlet–triplet energetic balance. This model is subsequently applied to determine the energy singlet–triplet resonance conditions in some prototypical organic molecules used in TTA-UC applications. This model also permits to study the mechanical response of singlet fission, which involves the reverse conversion of a singlet excitation into two triplet excitations, nevertheless we focus here in the TTA-UC to illustrate the method implementation.

The model provides therefore a practical computational protocol for identifying mechanically accessible structures with improved upconversion characteristics. This strategy may support the rational design of mechanoresponsive chromophores and materials in which optical behavior can be actively regulated by mechanical stress.

## Methodology

For all the organic TTA-UC emitters studied the calculations were carried out in the gas phase employing the M06–2X functional and the 6–31+G­(d,p) basis set using the Gaussian 16 program.^28^

The computational protocol began with the geometry optimization of each emitter in its ground electronic state (*S*_0_). The lowest-energy excited states, singlet (*S*_1_) and triplet (*T*_1_), were optimized independently employing the Tamm–Dancoff approximation (TDA) to time-dependent density functional theory, which helps mitigate excited-state instabilities particularly triplet instabilities, which can arise in conventional TD-DFT.^29^ Before the geometry optimization of T_1_ and *S*_1_, the stability of the ground-state wave function used as the reference for the TDA calculation was verified, and a stable solution was obtained in all cases. Frequency calculations without symmetry constraints were performed at the optimized *S*_0_, *S*_1_, and *T*_1_ geometries; the absence of imaginary frequencies confirmed that the resulting structures corresponded to minima on their respective potential energy surfaces. The optimized geometries and their zero-point vibrational energy corrections were also used to calculate the corresponding 0–0 transition energies, for comparison with the available experimental band-origin energies (see the Supporting Information).

The characterization of the *T*_1_ electronic state at its optimized geometry was carried out using the Multiwfn program,^30^ including natural transition orbital (NTO) analysis,^31^ HOMO–LUMO frontier orbital inspection, and electron–hole pair localization to characterize the excitation in each emitter. In addition to its independent geometry optimization, the *S*_1_ state was also evaluated vertically at the optimized *T*_1_ geometry and characterized using the same NTO and hole–electron analyses performed with Multiwfn (see the Supporting Information).

## Results

Here we present an algorithm to achieve mechanical control of the singlet–triplet resonance required for efficient triplet–triplet annihilation upconversion (TTA-UC). The central goal is to find the molecular structure under the action of an external mechanical force so that the vertical excitation energies satisfy the spectroscopic condition, i.e., the singlet excitation equals two times the triplet excitation, i.e., *E*_*S*_1__ – *E*_*S*_0__ = 2­(*E*_*T*_1__ – *E*_*S*_0__), while keeping the required force as small as possible. Without loss of generality, we focus on the lowest relevant excited states *S*_1_ and *T*_1_ (see Scheme 1).

**Scheme 1:**
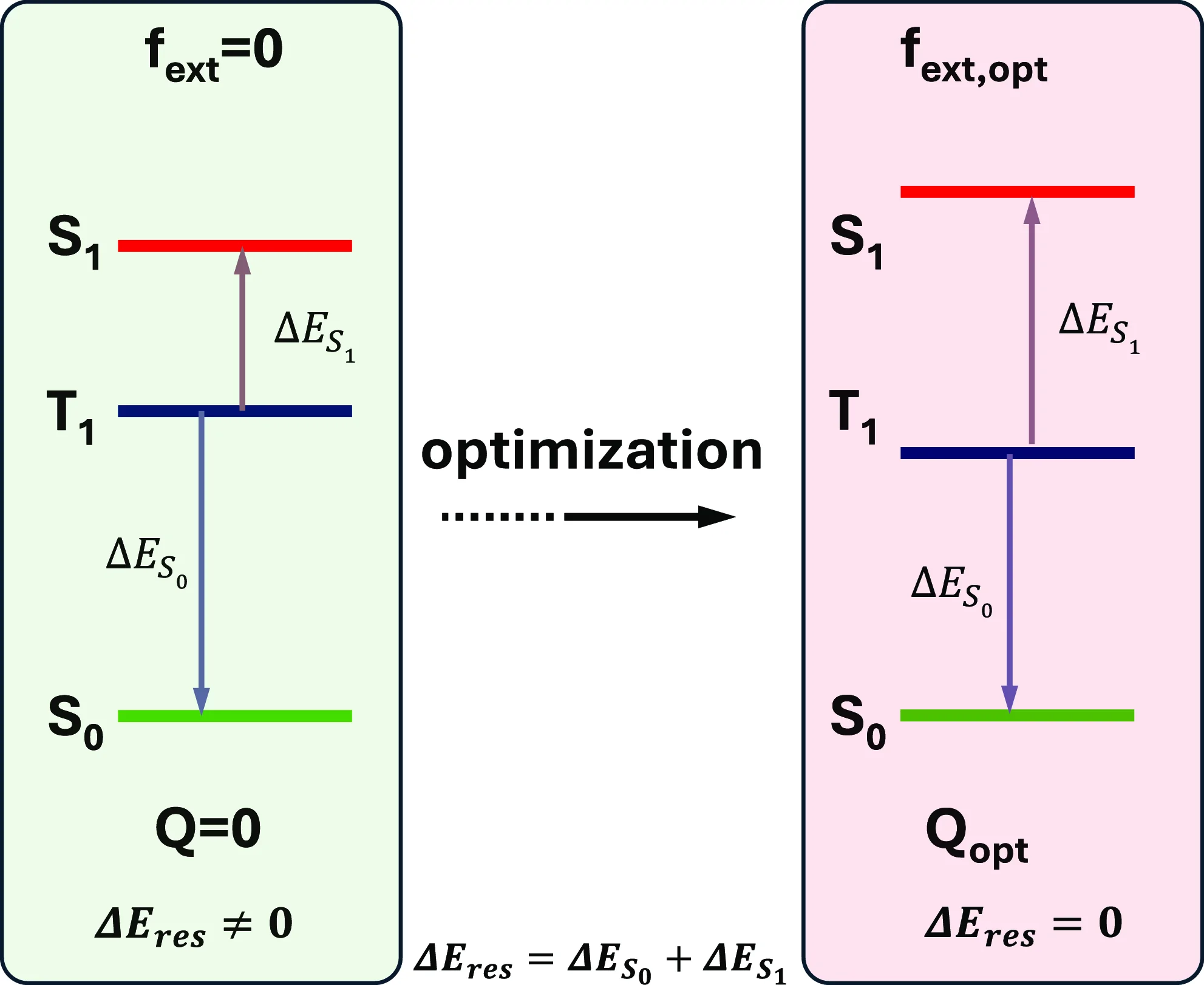
Scheme of Energy Levels and Resonance Condition, *E*_*S*_1__ – *E*_*S*_0__ = 2­(*E*_*T*_1__ – *E*_*S*_0__), in the Optimization Process

In the present formulation, the working resonance quantity is defined as Δ*E*_res_ = Δ*E*_*S*_0__ + Δ*E*_*S*_1__, where Δ*E*_*S*_0__ = *E*_*S*_0__ – *E*_*T*_1__ and Δ*E*_*S*_1__ = *E*_*S*_1__ – *E*_*T*_1__; exact resonance is reached for Δ*E*_res_ = 0, equivalent to the usual condition *E*_*S*_1__ – *E*_*S*_0__ = 2­(*E*_*T*_1__ – *E*_*S*_0__).

We then derive a constrained minimum-force update within a local quadratic description of the *T*_1_ surface and a linearized treatment of the relevant energy gaps, and finally describe the iterative workflow used to relinearize and converge to the exact resonance condition.

As mentioned above, implementation of the procedure for singlet fission is also possible. It could be possible to adapt the resonance condition to singlet fission by imposing the correct energy balance between states (i.e., a singlet state forming two triplet states): *E*_*S*_1__ + *E*_*S*_0__ = 2*E*_*T*_1__. Both processes are favored under different conditions, *E*_*S*_1__ + *E*_*S*_0__ < 2*E*_*T*_1__ favors exothermic TTA, while *E*_*S*_1__ + *E*_*S*_0__ > 2*E*_*T*_1__ favors exothermic singlet fission. Therefore, the proposed approach may be relevant to the mechanical tuning of both processes, TTA and singlet fission.

Additionally, another application of the algorithm is the mechanical tuning of the singlet–triplet gaps in thermally activated delayed fluorescence. This would imply controlling the *S*_1_–*T*_1_ energy gap.

Despite these possible extensions to other multistate tuning problems, here we focus on TTA-UC to illustrate the methodological implementation.

### Algorithm for Mechanical Resonance with Minimum Force

#### The Coordinate System and Local Potential-Energy Expansions

All nuclear degrees of freedom are described using the normal modes of the *T*_1_ electronic state

1 $$Q={\left(Q_{1},...,Q_{N}\right)}^{T}$$

with **Q** = 0 at the current reference geometry (initially the *T*_1_ minimum). At each iteration, the *T*_1_ PES is represented locally by a quadratic expansion

2 $$E_{T_{1}}\;\left(Q\right)=E_{T_{1}}\;\left(0\right)+Q^{T}g_{T_{1}}+\frac{1}{2}\;Q^{T}K_{T_{1}}Q$$

where **g**_*T*_1__ and **K**_*T*_1__ are, respectively, the gradient and Hessian of *T*_1_ in the chosen normal-coordinate basis. In these coordinates **K**_*T*_1__ is diagonal,

3 $$K_{T_{1}}=\mathrm{diag}\;\left(k_{T_{1},1},...,k_{T_{1},N}\right)$$

The PESs *S*_0_ and *S*_1_ are approached to have the same Hessian as *T*_1_ (linear approximation in the energy gaps),

4 $$K_{S_{0}}=K_{S_{1}}=K_{T_{1}}$$

so that

5 $$E_{S_{0}}\;\left(Q\right)=E_{S_{0}}\;\left(0\right)+Q^{T}g_{S_{0}}+\frac{1}{2}\;Q^{T}K_{T_{1}}Q$$

6 $$E_{S_{1}}\;\left(Q\right)=E_{S_{1}}\;\left(0\right)+Q^{T}g_{S_{1}}+\frac{1}{2}\;Q^{T}K_{T_{1}}Q$$

A constant external force **f**_ext_ is introduced in the same coordinate system, producing mechanical work

7 $$W=-Q^{T}f_{ext}$$

and an effective energy *E*_{*S*_0_,*S*_1_,*T*_1_}_(**Q**) – **Q** ^*T*^ **f**_ext_ for each electronic state.

#### Spectroscopic Resonance Condition with Minimal Force Magnitude

In the implementation, the relevant vertical energy gaps are defined relative to *T*_1_

8 $$\Delta E_{S_{0}}\;\left(Q\right)\equiv E_{S_{0}}\;\left(Q\right)-E_{T_{1}}\;\left(Q\right)$$

9 $$\Delta E_{S_{1}}\;\left(Q\right)\equiv E_{S_{1}}\;\left(Q\right)-E_{T_{1}}\;\left(Q\right)$$

Under the local quadratic model, and assuming same Hessians, these gaps become linear in the normal coordinates in a first approach

$$\Delta E_{S_{0}}\;\left(Q\right)=\Delta E_{S_{0}}\;\left(0\right)+Q^{T}\;\left(g_{S_{0}}-g_{T_{1}}\right)$$ 10

11 $$\Delta E_{S_{1}}\;\left(Q\right)=\Delta E_{S_{1}}\;\left(0\right)+Q^{T}\;\left(g_{S_{1}}-g_{T_{1}}\right)$$

We then define the working resonance quantity as

12 $$\Delta E_{res}\;\left(Q\right)\equiv \Delta E_{S_{0}}\;\left(Q\right)+\Delta E_{S_{1}}\;\left(Q\right)$$

so that

13 $$\Delta E_{res}\;\left(Q\right)=\Delta E_{res}\;\left(0\right)+Q^{T}c$$

with

14 $$\Delta E_{res}\;\left(0\right)=\Delta E_{S_{0}}\;\left(0\right)+\Delta E_{S_{1}}\;\left(0\right)$$

15 $$c\equiv g_{S_{0}}+g_{S_{1}}-2g_{T_{1}}$$

The objective is to find a resonant condition, which is reached when: Δ*E*_res_ (**Q**) = 0. Nevertheless, for practical purposes, it is possible to find intermediate conditions in order to keep the PESs approximations valid. Therefore, the condition can be formulated as

16 $$\Delta E_{res}\;\left(Q\right)=t$$

Being *t* a target energy, where exact singlet–triplet resonance corresponds to *t* = 0. In that case

17 $$E_{S_{1}}+E_{S_{0}}-2E_{T_{1}}=0$$

which is equivalent to the usual condition

18 $$E_{S_{1}}-E_{S_{0}}=2\;\left(E_{T_{1}}-E_{S_{0}}\right)$$

For any chosen displacement **Q**, the external force required to make *T*_1_ stationary at that point is obtained from the condition ∇*E*_*T*_1__(**Q**) = 0

19 $$f_{ext}\;\left(Q\right)=g_{T_{1}}+K_{T_{1}}Q$$

*T*_1_ is the state chosen as the reference state, therefore assuming that the emitter is relaxed in this state before triplet conversion.

Hence, the optimization problem is to find a displacement vector **Q** such that

20 $$\Delta E_{res}\;\left(Q\right)=t$$

while minimizing ∥**f**_ext_(**Q**)∥^2^. The corresponding Lagrangian function is

21 $$L\left(Q\right)={∥f_{ext}\;\left(Q\right)∥}^{2}+\lambda \left[\Delta E_{\mathrm{res}}\;\left(Q\right)-t\right]$$

with

22 $${∥f_{ext}\;\left(Q\right)∥}^{2}={\left(g_{T_{1}}+K_{T_{1}}Q\right)}^{T}\left(g_{T_{1}}+K_{T_{1}}Q\right)=g_{T_{1}}^{T}g_{T_{1}}+2g_{T_{1}}^{T}K_{T_{1}}Q+Q^{T}K_{T_{1}}^{2}Q$$

In this way, the central magnitude to be optimized is the external force magnitude, rather than energy as for instance in restrained energy optimization algorithms.

Stationarity with respect to **Q** gives

23 $$\nabla_{Q}L\;\left(Q\right)=2K_{T_{1}}^{2}Q+2K_{T_{1}}g_{T_{1}}+\lambda \;c=0$$

leading to the optimal -minimum force magnitude- displacement, **Q**_opt_, under the linear approach in the vertical excitation energies.

24 $$Q_{opt}=-K_{T_{1}}^{-2}\;\left(\frac{\lambda }{2}c+K_{T_{1}}g_{T_{1}}\right)$$

The associated optimal external force eq 19 becomes

25 $$f_{ext,opt}=g_{T_{1}}+K_{T_{1}}Q_{opt}=-\frac{\lambda }{2}K_{T_{1}}^{-1}c$$

Substituting **Q**_opt_ into the constraint yields

26 $$\Delta E_{res}\;\left(Q_{opt}\right)=\Delta E_{res}\;\left(0\right)+Q_{opt}^{T}c=t$$

Or

27 $$\Delta E_{res},\;\left(Q_{opt}\right)=\Delta E_{res}\;\left(0\right)-\beta -\lambda \Delta E_{SF,mec}=t$$

where

28 $$\beta \equiv g_{T_{1}}^{T}K_{T_{1}}^{-1}c$$

and

29 $$\Delta E_{res,mec}\equiv \frac{1}{2}\;c^{T}K_{T_{1}}^{-2}c$$

Therefore,

30 $$\lambda =\frac{\Delta E_{res}\;\left(0\right)-t-\beta }{\Delta E_{res,mec}}$$

If the reference geometry is an exact *T*_1_ minimum, then **g**_*T*_1__ = 0 and the β term vanishes. Additionally, the above expression is formulated for a general target value *t*. Nevertheless, exact singlet–triplet resonance is recovered for *t* = 0.

Because **K**_*T*_1__ is diagonal in normal coordinates, **K**_*T*_1__^–1^ and **K**_*T*_1__^–2^ are straightforwardly computed, which makes the update numerically inexpensive.

It has to be noted that the present approach does not explicitly consider competitive channels involving higher energy triplet states or quintet states, or in general the spin evolution of the triplet pair in the TTA process. Nevertheless, additional energetic requirements could be introduced as additional constraints maintaining the same mechanical optimization procedure.

#### Description of the Iterative Optimization Scheme

The above solution is exact within the local quadratic/linear model defined at the current reference geometry, where **Q** = **0** by definition. Because **g**_*T*_1__, **g**_*S*_0__, **g** *_S_*__1__, Δ*E*_*S*_0__(**0**), Δ*E*_*S*_1__(**0**) and, if required, **K**_*T*_1__ change with geometry, the optimization is carried out iteratively through successive re-evaluation of the local model at updated reference structures. At each iteration, the same constrained minimum-force problem is solved using the quantities computed at the current geometry:

1. Initialization. Start from an initial reference geometry and define **Q** = **0** at that structure, using the normal-mode basis of the *T*_1_ state associated with the current point.
2. Local electronic-structure step. At the current reference geometry, compute **K**_*T*_1__, **g**_*T*_1__, **g**_*S*_0__, **g**_*S*_1__ and the electronic energies *E*_*T*_1__(**0**), *E*_*S*_0__(**0**), and *E*_*S*_1__(**0**), or equivalently the quantities

31 $$\Delta E_{S_{0}}\;\left(0\right)=E_{S_{0}}\;\left(0\right)-E_{T_{1}}\;\left(0\right)$$

and

32 $$\Delta E_{S_{1}}\;\left(0\right)=E_{S_{1}}\;\left(0\right)-E_{T_{1}}\;\left(0\right)$$

3. Construction of the resonance condition. Form the vector

33 $$c=g_{S_{0}}+g_{S_{1}}-2g_{T_{1}}$$

and evaluate

34 $$\Delta E_{res}\;\left(0\right)=\Delta E_{S_{0}}\;\left(0\right)+\Delta E_{S_{1}}\;\left(0\right)$$

The resonance constraint is imposed as

35 $$\Delta E_{res}\;\left(Q\right)=t$$

where exact singlet–triplet resonance corresponds to *t* = 0.

4. Solution of the constrained minimum-force problem. Using the local quadratic/linear model, evaluate

36 $$\Delta E_{res,mec}\equiv \frac{1}{2}c^{T}K_{T_{1}}^{-2}c$$

and

37 $$\beta \equiv g_{T_{1}}^{T}K_{T_{1}}^{-1}c$$

Then obtain

38 $$\lambda =\frac{\Delta E_{res}\;\left(0\right)-t-\beta }{\Delta E_{res,mec}}$$

followed by the optimal displacement **Q**_opt_ and the corresponding optimal external force **f**_ext,opt_.

5. Geometry update. Transform **Q**_opt_ from the *T*_1_ normal-coordinate representation to Cartesian coordinates and generate the updated molecular geometry.
6. Recentering of the local model. Define the updated geometry as the new reference point, so that **Q** = **0** is re-established at the displaced structure, and construct a new local quadratic/linear model at that geometry.
7. Iteration and convergence. Repeat steps 2–6 until the resonance condition is satisfied within a chosen tolerance

39 $$\left|\Delta E_{res}\;\left(0\right)-t\right|<\varepsilon$$

For exact singlet–triplet resonance, this reduces to

40 $$\left|\Delta E_{res}\;\left(0\right)\right|<\varepsilon$$

In this way, the optimization is achieved through the successive application of locally defined constrained minimum-force updates, each followed by a redefinition of the reference geometry and a re-evaluation of the electronic-structure quantities entering the model.

### Implementation of the Algorithm in Case-Study Systems

To validate the applicability and robustness of the developed algorithm, we applied our methodology to three chromophores exhibiting different degree of structural flexibility and mechanical response. First, 1,4-bis­(phenylethynyl)­benzene (BPEB) was selected given its relevance as emitter in TTA-UC systems and the availability of experimental reference data.^32,33^ We also studied anthracene (An),^34,35^ chosen specifically because it is a planar and rigid molecule that does not undergo torsional deformation under relatively small forces. Finally, we analyzed 9,10-diphenylanthracene (DPA),^36,37^ which presents a distinct behavior as it is a less rigid system than anthracene and undergoes a subtle concave deformation. The selection of this set of molecules allows us to explore how the optimal external force required to reach the resonance condition depends on the different molecular characteristics, including susceptibility to torsional bending and bond stretching.

Of course, the proposed procedure permits to identify the optimal forces controlling the TTA-UC process, but it does not describe the dynamics of the system. Explicit inclusion of the mechanical forces in the force field would be necessary to describe the nonadiabatic dynamics of the systems.

#### 1,4-Bis­(phenylethynyl)­benzene

In BPEB, both the *T*_1_ and *S*_1_ states are ππ* excitations with dominant HOMO→LUMO character and no significant charge-transfer character (see Figure 1 and Supporting Information for details). *S*_1_ lies at 3.48 eV and is strongly allowed (bright), while *T*_1_ is the corresponding lower-lying triplet of the same orbital nature. The *S*_1_ transition is polarized along the long molecular axis, along which the hole and electron are delocalized.

**Figure 1:**
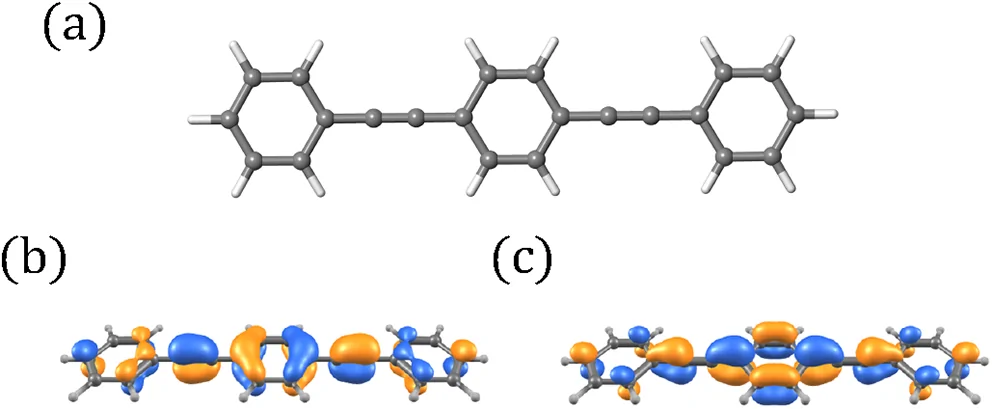
(a) *T*_1_ equilibrium structure of BPEB. (b) HOMO and (c) LUMO frontier orbitals. Isosurfaces are plotted at a contour value of 0.04 au.

Comparison of the theoretical results with the 0–0 transition energies of BPEB for both the *S*_1_ and *T*_1_ band origins,^32,33^ show a reasonable agreement, with differences ∼0.1 eV between the experimental and computational results (see Supporting Information for details). Upon confirming the accuracy of the method, it was employed to obtain the *S*_0_–*T*_1_ and *S*_1_–*T*_1_ vertical energy gaps at the *T*_1_ equilibrium geometry. getting −52.1 and 28.1 kcal·mol^–1^, respectively (Δ*E*_res_ = −23.9 kcal·mol^–1^).

As depicted in Figure 2, three independent routes were explored for BPEB, obtained by setting *t* values of +1.0, +6.0 and 0.0 kcal mol^–1^. All three converge to points exhibiting negligible differences, showing a consistent stabilization along the internal coordinates q_1_ (stretching) and q_2_ (bending; see Supporting Information for the exact definition) upon reaching the resonance condition.

**Figure 2:**
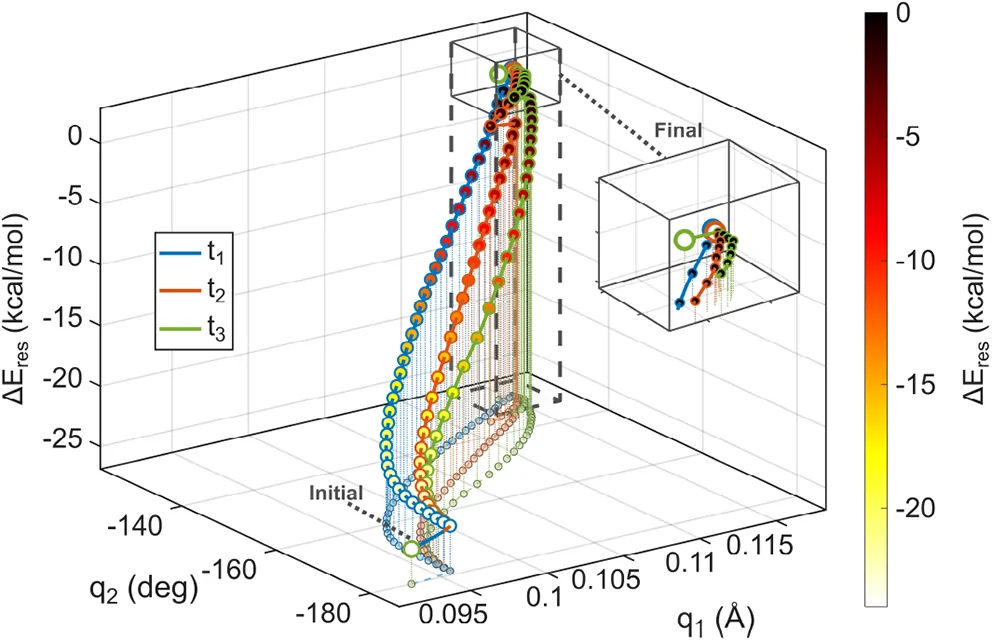
Representation of the resonance energy Δ*E*_res_ as a function of the internal coordinates q_1_ (stretching, Å) and q_2_ (bending deformation, degrees) for BPEB. The three explored routes shown have been obtained for *t* values of +1.0 kcal·mol^–1^ (*t*_1_), +6.0 kcal·mol^–1^ (*t*_2_), 0.0 kcal·mol^–1^ (*t*_3_).

As shown in Table 1, route *t*_1_ required approximately 41 cycles to reach this condition, while routes *t*_2_ and *t*_3_ converged in 39 cycles, respectively. In all cases the computed optimal force has a magnitude of ca. 0.14 nN (see Table 1).

**Table 1:** Number of Optimization Cycles, Resonance Energy (kcal·mol^–1^), External Force (nN), and Mechanical Sensitivity (kcal·mol^–1^·nN^–1^) for Each Explored Route in BPEB

	N° of cycles	Δ*E*_res_	*f*_ext_	σ
*t*_1_	41	0.003	0.14	171.4
*t*_2_	39	–0.008	0.15	163.4
*t*_3_	39	–0.007	0.14	170.1

In order to quantify the mechanical response of the molecular system to reach the resonance condition, it is possible to define the mechanical sensitivity σ as

$$\sigma \equiv \frac{\Delta E_{res}\;\left(0\right)}{\left|f_{ext}\right|}$$

i.e., the ratio between Δ*E*_res_ (**0**), the energy variation to reach the resonance condition, and the optimal force magnitude needed to reach the resonance condition. This magnitude provides the mechanical response of the chromophore for the resonance condition, with high values indicating feasible mechanical control and low values pointing out that external forces have a small effect in tuning the relative energies of the electronic states.

In the BPEB case, σ is ca. 170 kcal·mol^–1^·nN^–1^, indicating that the relative energies of the electronic states involved in TTA-UC are highly sensitive to mechanical perturbations. A relatively small force (in order of tens of pN) is enough to reach the resonance condition.

Figure 3 shows the structural comparison between the initial *T*_1_ equilibrium structure and that fulfilling the resonance condition using a molecular alignment.^38^ As can be seen, the dominant deformation is torsional, with the internal coordinate *q*_2_ varying from −180.0° to −129.4°, adopting a horseshoe-like conformation and bending the molecule in the middle. This is further supported by the optimal external force vectors, where a predominantly torsional character is observed (see Figure 4).

**Figure 3:**
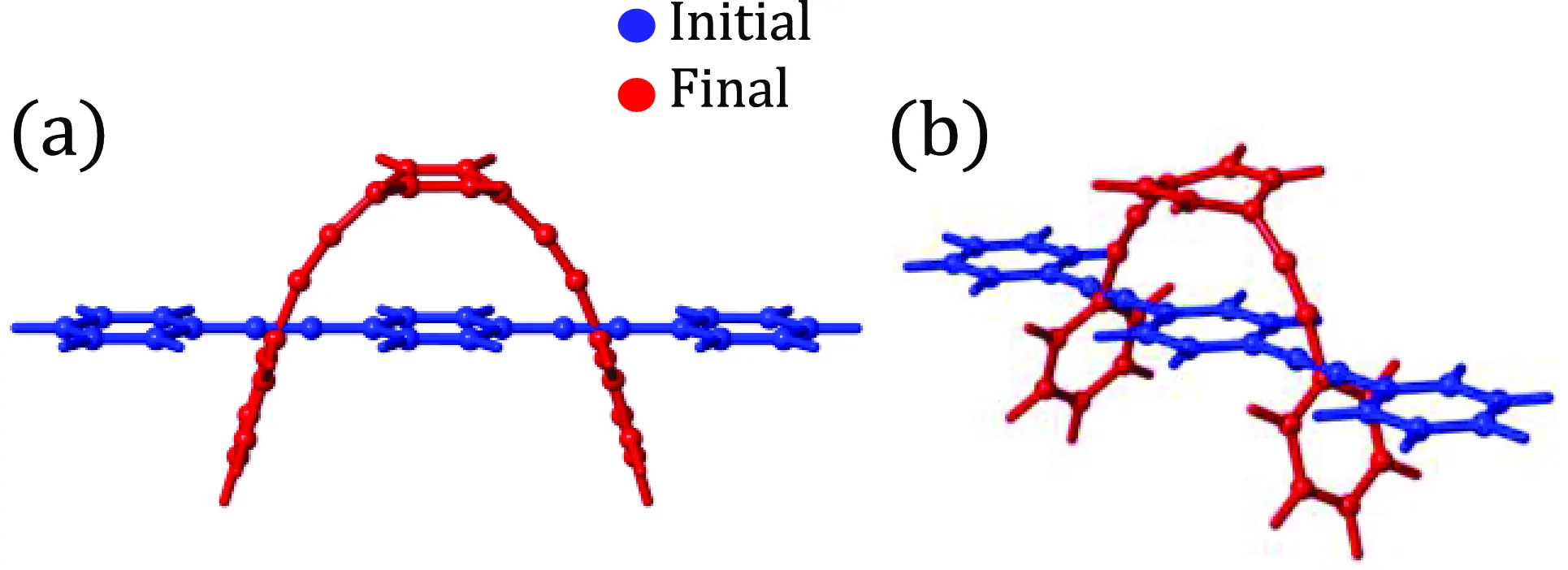
Structural comparison between the initial (blue) and final (red) *T*_1_ structure fulfilling the resonance condition Δ*E*_res_ = 0.0 kcal·mol^–1^ of BPEB shown from (a) frontal and (b) lateral views.

**Figure 4:**
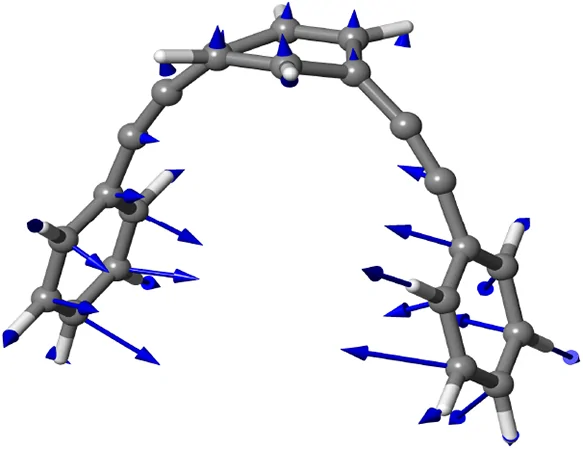
Distorted *T*_1_ equilibrium structure of BPEB at the resonance condition Δ*E*_res_ = 0 kcal·mol^–1^. Blue vectors represent the optimal external forces acting on each atom.

#### Anthracene

As shown in Figure 5, the *T*_1_ state of An is also a ππ* excitation with dominant HOMO→LUMO character (see Supporting Information for further details). Likewise, *S*_1_ exhibit a ππ* character with a dominant HOMO→LUMO excitation. In line with BPEB, neither *T*_1_ nor *S*_1_ shows significant charge-transfer character.

**Figure 5:**
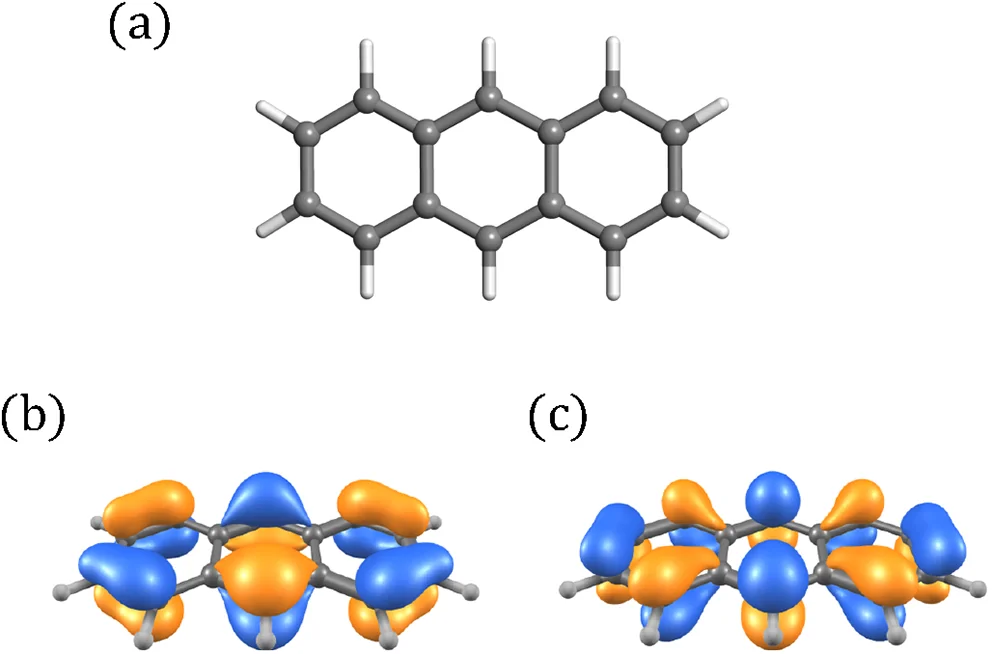
(a) *T*_1_ equilibrium structure of An. (b) HOMO and (c) LUMO frontier orbitals. Isosurfaces are plotted at a contour value of 0.04 au.

The reliability of the method was again confirmed by comparing the theoretical results with the experimental gas-phase 0–0 transition energies of the *S*_1_ and *T*_1_ excited electronic states of An.^34,35^ The method successfully reproduced the band origins of both the *S*_1_ and *T*_1_ states within ∼0.14 eV (see Supporting Information for further details). Therefore, we used it to evaluate the *S*_0_–*T*_1_ and *S*_1_–*T*_1_ vertical energy gaps at the *T*_1_ equilibrium geometry, obtaining −40.8 and 36.5 kcal·mol^–1^, respectively (Δ*E*_res_ = −4.3 kcal·mol^–1^)

Unlike BPEB, An is a planar and rigid molecule that does not undergo torsional deformation under relatively small forces. This is apparent when applying the algorithm to this molecule. Two routes were explored: *t*_1_ (+1.0 kcal·mol^–1^) and *t*_2_ (0.0 kcal·mol^–1^). As shown in Figure 6, both routes converge to the same optimal point with no evidence of torsional deformation. As summarized in Table 2, route *t*_1_ required 5 steps to reach the resonance condition, while *t*_2_ did so in a single step. In both cases, the forces stabilize at approximately 2.0 nN with a sensitivity of around 2.3 kcal·mol^–1^·nN^–1^.

**Figure 6:**
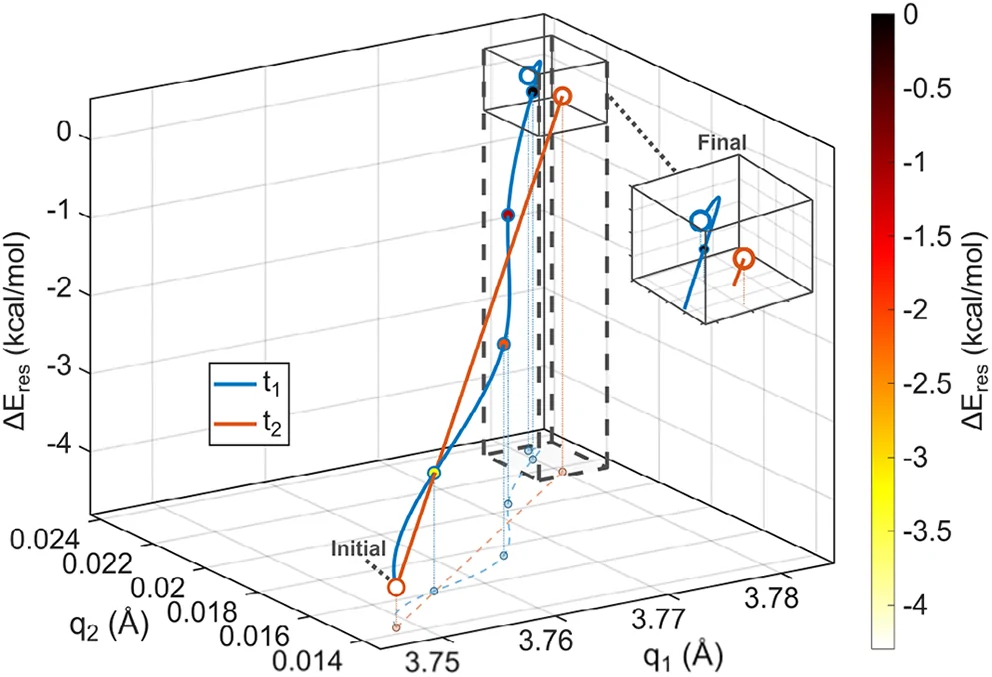
Representation of the resonance energy Δ*E*_res_ as a function of the internal coordinates q_1_ and q_2_ (stretching, Å) for An. The two explored routes correspond to *t* values of +1.0 kcal·mol^–1^ (*t*_1_), and 0.0 kcal·mol^–1^(*t*_2_).

**Table 2:** Number of Optimization Cycles, Resonance Energy (kcal·mol^–1^), External Force (nN), and Mechanical Sensitivity (kcal·mol^–1^·nN^–1^) for Each Explored Route in An

	N° of cycles	Δ*E*_res_	*f*_ext_	σ
*t*_1_	5	<0.001	1.88	2.3
*t*_2_	1	0.006	1.96	2.3

As shown in Figure 7, the comparison between the initial *T*_1_ geometry and the final distorted geometry reveals that both are structurally very similar, confirming that An, given its rigidity, undergoes minimal structural changes upon reaching the resonance condition. This is consistent with the low sensitivity values observed (Table 2). Furthermore, this is supported by the optimal external force vectors shown in Figure 8, which are symmetrically distributed over the molecular plane and oriented primarily along the C–C and C–H bonds. This shows that the mechanical response of An is exclusively of stretching nature, with no torsional component in the process investigated.

**Figure 7:**
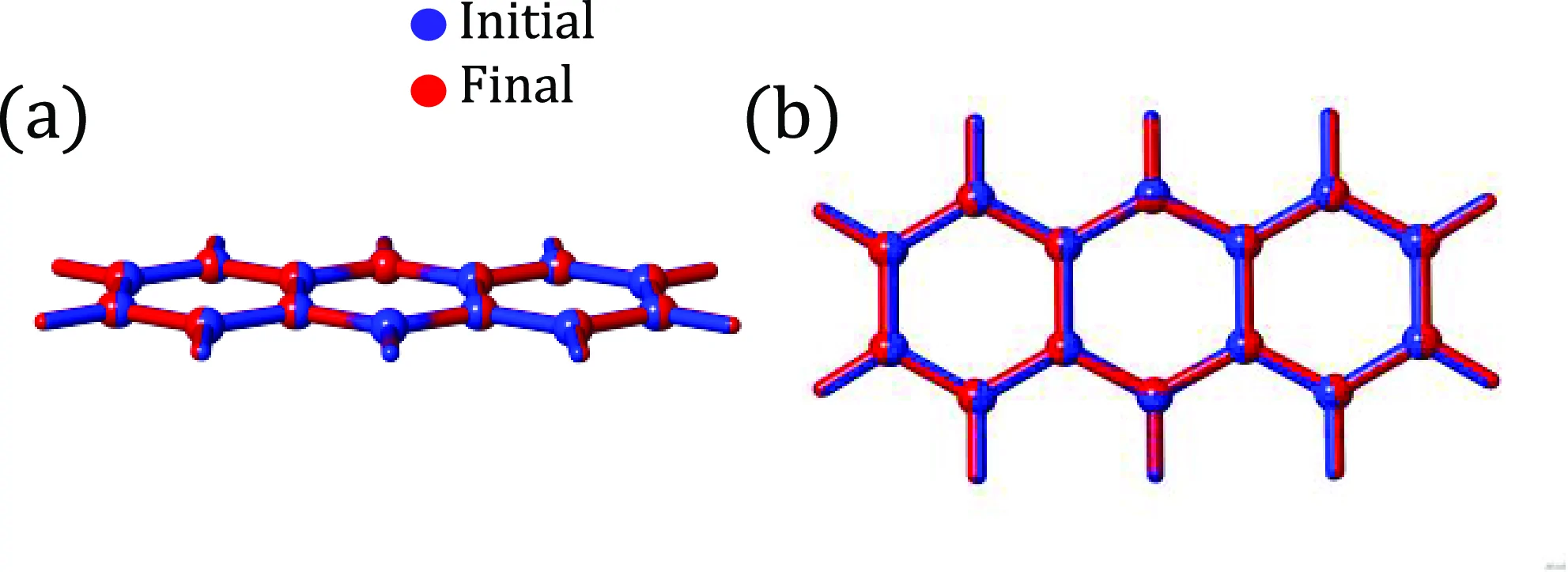
Structural comparison between the initial (blue) and final (red) *T*_1_ structure fulfilling the resonance condition Δ*E*_res_ = 0 kcal·mol^–1^ of An shown from (a) frontal and (b) lateral views.

**Figure 8:**
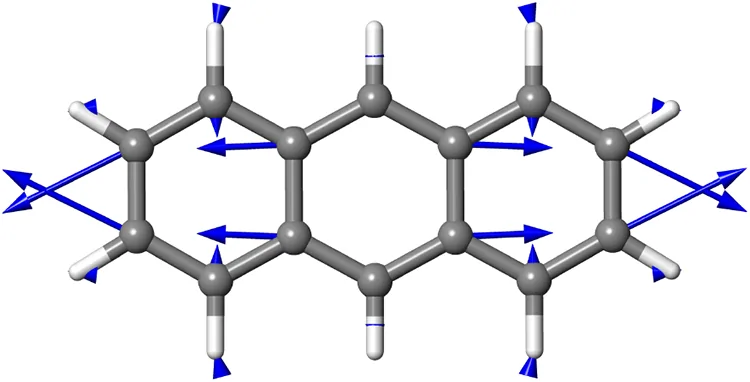
Distorted *T*_1_ equilibrium structure of An at the resonance condition Δ*E*_res_ = 0.0 kcal·mol^–1^. Blue vectors represent the forces acting on each atom.

#### 9,10-Diphenylanthracene

As shown in Figure 9, the *T*_1_ state of DPA is a ππ* excitation localized on the anthracene core, with dominant HOMO→LUMO character (see Supporting Information for further details). Likewise, *S*_1_ is of the same nature: a dominant HOMO→LUMO excitation confined to the anthracene core, with no significant charge-transfer character. This optically bright state is located at 3.13 eV above the ground state.

**Figure 9:**
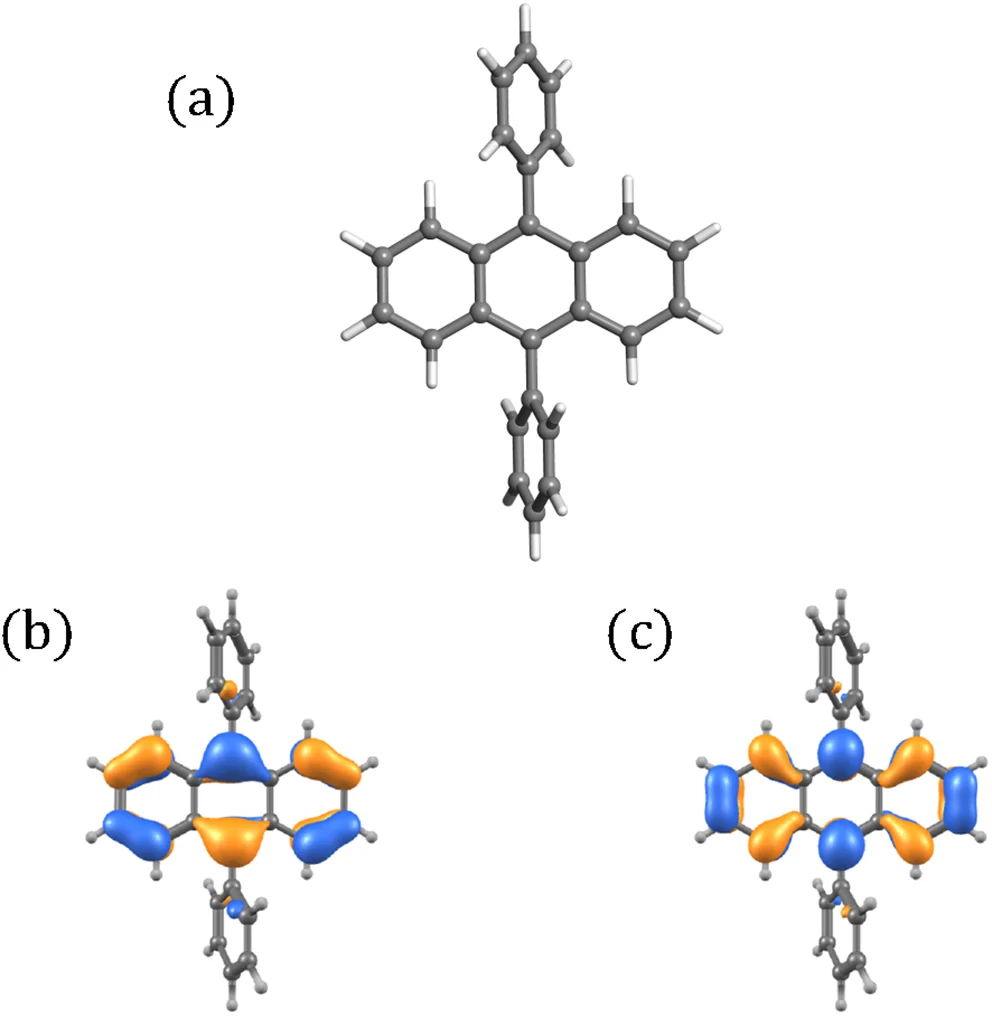
(a) Optimized *T*_1_ equilibrium structure of DPA. (b) HOMO and (c) LUMO frontier orbitals. Isosurfaces are plotted at a contour value of 0.04 au.

Like in the BPEB and An cases, we verified the ability of the method to characterize the electronic states of interest in DPA. Comparison of the theoretical results with the experimental gas-phase 0–0 transition energies of the *S*_1_ and *T*_1_ excited electronic states of DPA,^36,37^ shows that the method reproduces the band origin of *S*_1_ within 0.11 eV and that of *T*_1_ with 0.16 eV (see Supporting Information for details). As in the previous cases, the method was employed to evaluate the *S*_0_–*T*_1_ and *S*_1_–*T*_1_ vertical energy gaps at the *T*_1_ equilibrium geometry, obtaining −38.3 and 33.9 kcal·mol^–1^, respectively (Δ*E*_res_ = −4.4 kcal·mol^–1^).

Like in the An case, two trajectories were considered, defined by *t*_*1*_ (1.0 kcal mol^–1^) and *t*_*2*_ (0.0 kcal mol^–1^) and propagated along the internal stretching coordinates q_1_ and q_2_. As shown in Figure 10, both trajectories converge to the same point within approximately 12 cycles, reaching the resonance condition at an applied force of 0.18 nN, with a sensitivity of 24.1 kcal·mol^–1^·nN^–1^ (see Table 3). Unlike An, DPA undergoes a slight concave bending of its molecular backbone upon reaching resonance.

**Figure 10:**
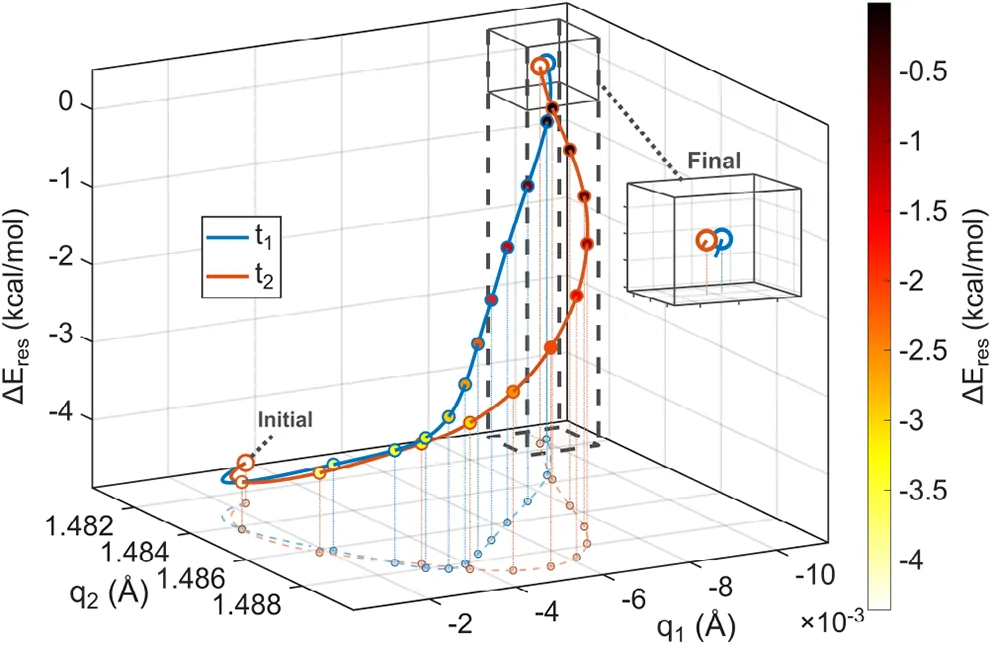
Representation of the resonance energy Δ*E*_res_ as a function of the internal coordinates q_1_ and q_2_ (stretching, Å) for DPA. The two explored routes correspond to *t*_1_ (+1.0 ), and *t*_2_ (0.0 kcal·mol^–1^).

**Table 3:** Number of Optimization Cycles, Resonance Energy (kcal·mol^–1^), External Force (nN), and Mechanical Sensitivity (kcal·mol^–1^·nN^–1^) for Each Explored Route in DPA

	N° of cycles	Δ*E*_res_	*f*_ext_	σ
*t*_1_	12	–0.031	0.18	23.4
*t*_2_	12	–0.011	0.18	24.1

Figure 11 shows the comparison between the initial *T*_1_ geometry and the final distorted geometry of DPA. As can be seen, the molecular distortions that lead to the resonance condition are a combination of out-of-plane bending of the central An moiety combined with the stretching of the lateral phenyl rings. A look at the external force vectors shown in Figure 12, where larger magnitude vectors are observed at the lateral phenyl rings pointing outward, consistent with a mixed mechanical response involving both stretching and torsional contributions, further illustrates this.

**Figure 11:**
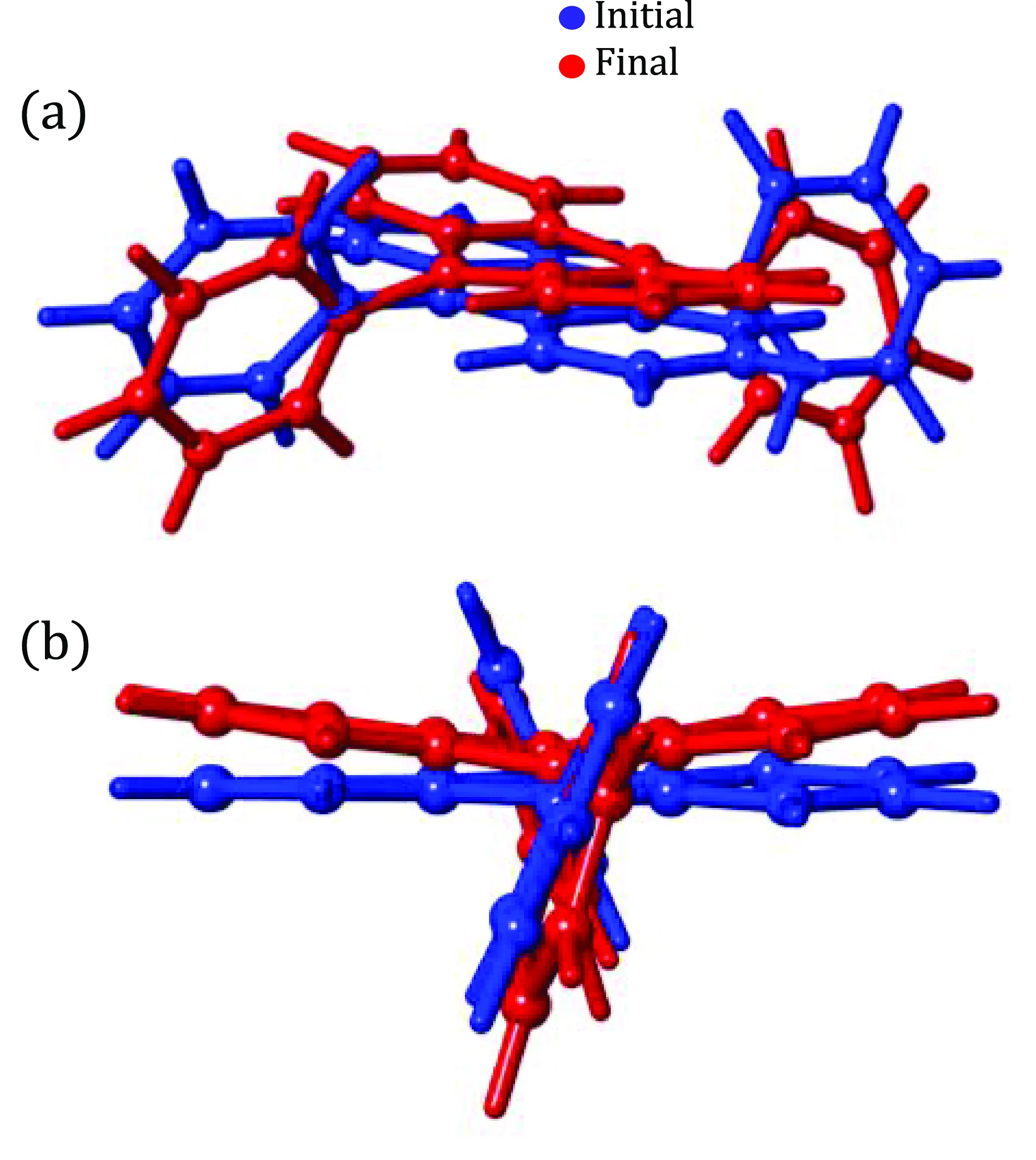
Structural comparison between the initial (blue) and final (red) *T*_1_ structure fulfilling the resonance condition Δ*E*_res_ = 0.0 kcal·mol^–1^ of DPA shown from (a) frontal and (b) lateral views.

**Figure 12:**
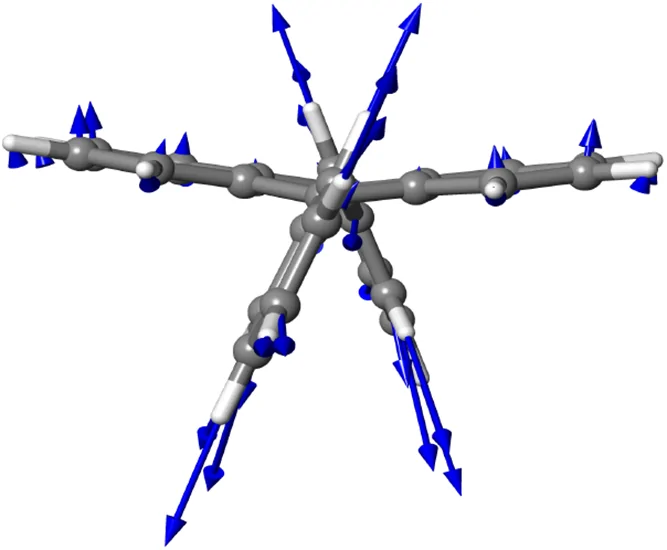
Distorted *T*_1_ geometry of DPA at the resonance condition Δ*E*_res_ = 0.0 kcal·mol^–1^. Blue vectors represent the internal forces acting on each atom.

Summarizing, the algorithm developed here has been tested with different triplet emitters, showing all of them different behavior and specific optimal external forces (and the consequent structural distortions) permitting the system to reach the resonance condition. The inclusion of an explicit environment (e.g., solvent model), instead of the isolated molecule, is also straightforward in the present algorithm, as the PES can be approached in the exact same way, where the energies, gradients and Hessian are determined now by the specific environment.

As has been pointed out above, using alternative resonance conditions with the exact algorithm scheme, it could also be possible to extend the study to singlet fission and eventually thermally activated delayed fluorescence. The model does not tackle with alternative processes like competitive deactivation channels, since the target is to identify the optimal forces controlling the resonance condition. These processes can be computationally studied for specific systems by including the found mechanical force in the force field.

## Conclusions

We have developed and implemented a novel computational algorithm designed to mechanically tune the singlet–triplet energetic resonance required for efficient TTA-UC. By formulating the problem as a constrained minimum-force optimization, our method iteratively identifies the optimal molecular geometry and the minimal external force vector needed to satisfy the target resonance condition.

The application of this algorithm to three selected emitters (BPEB, An and DPA) allows us to identify the optimal forces and the associated molecular structural changes that allow to efficiently modulate the energies of *S*_0_/*T*_1_/*S*_1_ states to reach the resonance condition. Our findings show that flexible systems like BPEB exhibit high mechanical sensitivity, achieving the necessary energetic balance under minimal external forces (in the range of hundreds of pN). This energetic tuning is mainly reached through symmetric torsional deformations that bend the molecule into a horseshoe-like conformation. For rigid systems, like An, the mechanical sensitivity is significantly lower. Given its planar rigidity, it reaches the resonance condition with much larger mechanical forces (approximately 2.0 nN), which were absorbed exclusively via in-plane bond stretching deformations without any torsional component. Finally, intermediate behavior systems like DPA exhibit a mixed mechanical response. While less rigid than An, its sensitivity is lower than that of BPEB, allowing it to adapt to target energies via a structural complex concave bending and also involving stretching coordinates mainly at the lateral phenyl rings.

It has to be noted that this approach can be easily extended to explore the mechanical control of singlet fission (i.e., by using *E*_*S*_1__ + *E*_*S*_0__ = 2*E*_*T*_1__) and to TADF by considering *S*_1_ equal to *T*_1_.

This methodology offers a practical computational strategy to quantify mechanical sensitivity and to identify mechanically accessible molecular structures with tuned upconversion properties. Furthermore, this algorithm also establishes a valuable predictive tool to support the rational design of novel mechanoresponsive chromophores to be used in the design of advanced materials with stress-tunable optical behavior.

## Supplementary Material



## Data Availability

The computational data supporting this work are openly available in Zenodo at DOI: 10.5281/zenodo.21536068. The code of the implemented algorithm is available at https://github.com/LMFrutos/Mechanical-TTA-UC

## Notes

Internal-Coordinate Definitions; stagewise Data of the Optimal-Force Resonance Procedure; two-Dimensional Resonance Trajectories; Hole–Electron Descriptors; optimized Cartesian Coordinates; resonance Cartesian Coordinates; and benchmarking against experimental 0–0 energies (PDF)
