# Supplementary material for: Mechanical Tuning of Singlet–Triplet Resonance for Efficient Triplet–Triplet Annihilation Upconversion
Source: J Chem Theory Comput. 2026 Sep 25;22(19):10381–90. doi: 10.1021/acs.jctc.6c01488 (PMC13671174; doi:10.1021/acs.jctc.6c01488)
Supplement: Supplementary file 1 [file ct6c01488_si_001.pdf]

Supporting Information

Mechanical Tuning of Singlet–Triplet Resonance for Efficient Triplet-Triplet Annihilation Upconversion

Edgard Miranda-Sáenz,[a,b] Pedro B. Coto,[a,*] and Luis Manuel Frutos[b,c*]

[a] Nanomaterials and Nanotechnology Research Center (CINN) Spanish National Research Council (CSIC) and Donostia International Physics Center (DIPC). Avenida de la Vega 4–6, El Entrego 33940, Spain. [b] Universidad de Alcalá, Departamento de Química Analítica, Química Física e Ingeniería Química, Grupo de Reactividad y Estructura Molecular (RESMOL), Alcalá de Henares 28805, Madrid, Spain. [c] Universidad de Alcalá, Instituto de Investigación Química "Andrés M. del Río" (IQAR), Alcalá de Henares 28805, Madrid, Spain.

Content

1. Internal-Coordinate Definitions **S3**
Figure S1. Atom numbering of BPEB S3
Figure S2. Atom numbering of anthracene S4
Figure S3. Atom numbering of DPA S5

2. Stagewise Data of the Optimal-Force Resonance Procedure **S6**
Table S1. BPEB, route t_1 .... S6
Table S2. BPEB, route t_2 .... S7
Table S3. BPEB, route t_3 .... S8
Table S4. Anthracene, route t_1 .... S9
Table S5. Anthracene, route t_2 .... S9
Table S6. DPA, route t_1 .... S9
Table S7. DPA, route t_2 .... S10

3. Two-Dimensional Resonance Trajectories **S11**
Figure S4. BPEB, routes t_1, t_2, and t_3 .... S11
Figure S5. Anthracene, routes t_1 and t_2 .... S11
Figure S6. DPA, routes t_1 and t_2 .... S12

4. Hole–Electron Descriptors **S13**
Table S8. Hole–electron descriptors of the T_1 states of the four emitters S13
Figure S7. Leading T_1 hole–electron NTO pairs of BPEB, anthracene, and DPA S14
Table S9. Hole–electron descriptors of the S_1 states of the four emitters S14
Figure S8. Leading S_1 hole–electron NTO pairs of BPEB, anthracene, and DPA S15

5. Optimized Cartesian Coordinates **S16**
Table S10. BPEB, S_0 optimized geometry S16
Table S11. BPEB, T_1 optimized geometry S16
Table S12. BPEB, S_1 optimized geometry S17
Table S13. Anthracene, S_0 optimized geometry S18
Table S14. Anthracene, T_1 optimized geometry S19
Table S15. Anthracene, S_1 optimized geometry S19
Table S16. DPA, S_0 optimized geometry S20
Table S17. DPA, T_1 optimized geometry S21
Table S18. DPA, S_1 optimized geometry S22

6. Resonance Cartesian Coordinates **S23**
Table S19. BPEB, resonance geometry obtained along route t_1 .... S23
Table S20. BPEB, resonance geometry obtained along route t_2 .... S23

Table S21. BPEB, resonance geometry obtained along route t_3 .... S24
Table S22. Anthracene, resonance geometry obtained along route t_1 .... S25
Table S23. Anthracene, resonance geometry obtained along route t_2 .... S26
Table S24. DPA, resonance geometry obtained along route t_1 .... S26
Table S25. DPA, resonance geometry obtained along route t_2 .... S27

7. Benchmarking against experimental 0-0 energies.......... S29
Table S26. Calculated vs. experimental 0-0 energies (E_{00}, eV) of BPEB.......... S29
Table S27. Calculated vs. experimental 0-0 energies (E_{00}, eV) of Anthracene.......... S29
Table S28. Calculated vs. experimental 0-0 energies (E_{00}, eV) of DPA.......... S30

8. References S31

1. Internal-Coordinate Definitions

1.1. Case BPEB

For BPEB, the internal coordinate q_1 represents the normalized quinoidal coordinate, computed as $q_1 = \frac{1}{2}(r_{C6-C10} + r_{C3-C1} - r_{C2-C6} - r_{C7-C3})$, capturing the asymmetric redistribution of the most active bonds in the molecular backbone. q_2 represents the torsional deformation, defined as $q_2 = \frac{1}{2}(\varphi_1 - \varphi_2)$, where $\varphi_1 = \angle(C2-C6-C7-C14)$ and $\varphi_2 = \angle(C6-C2-C3-C34)$.

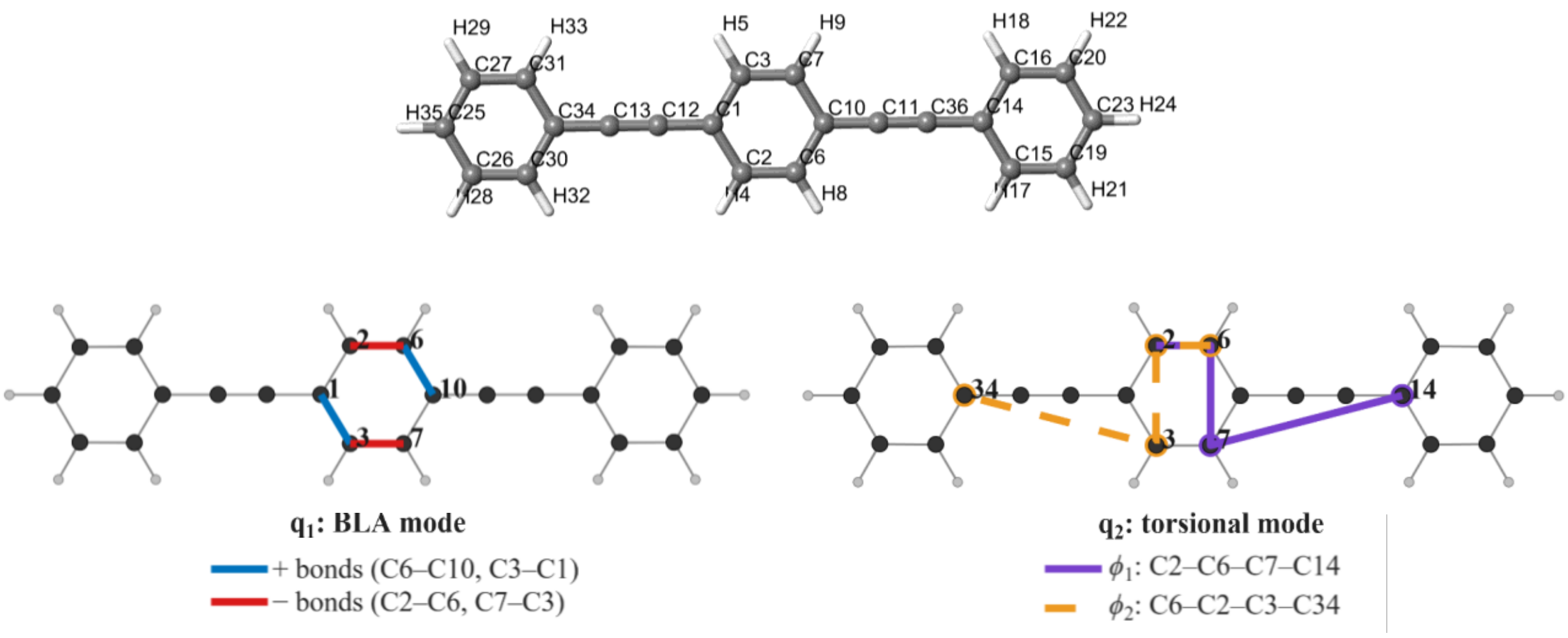

Figure S1. Atom numbering of BPEB used throughout this work, corresponding to the standard orientation of the T_1-optimized geometry.

1.2. Case Antharecene

q₁ represents the global symmetric mode, computed as $q_1 = \frac{1}{\sqrt{7}}(r_{C3-C2} + r_{C4-C5} + r_{C5-C6} + r_{C1-C8} + r_{C19-C9} + r_{C11-C14} + r_{C13-C12})$, capturing the total expansion of the molecular backbone. q₂ represents the antisymmetric quinoidal mode, defined as $q_2 = \frac{1}{2}(r_{C3-C2} + r_{C5-C6} - r_{C1-C8} - r_{C13-C12})$.

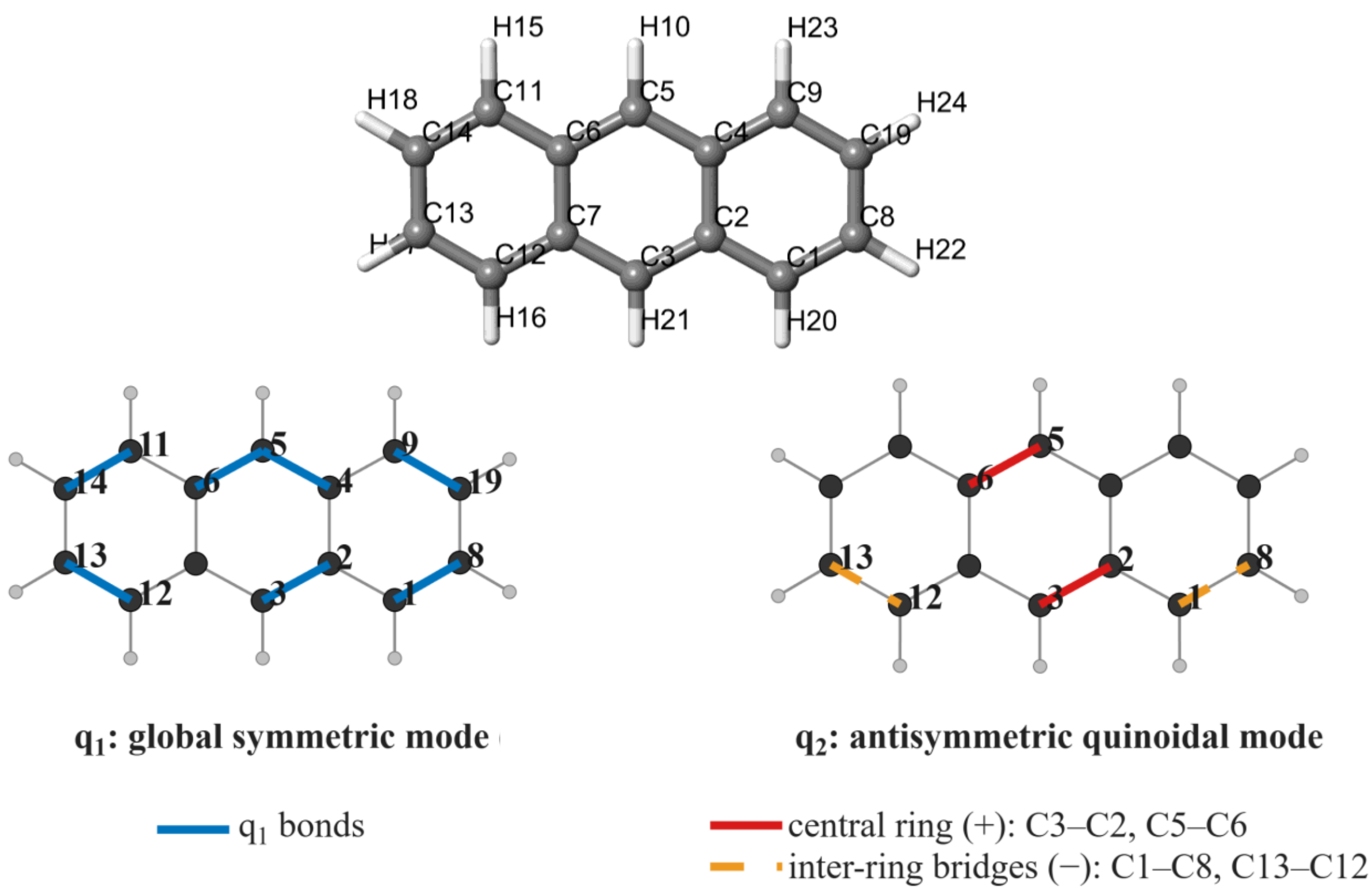

Figure S2. Atom numbering of Anthracene used throughout this work, corresponding to the standard orientation of the T_1-optimized geometry.

1.3. Case DPA

q_1 represents the Kekulé/quinoidal mode of the central anthracene ring, computed as the difference between the mean fusion-bond length and the mean meso-bond length, $q_1 = ½[r(C2 - C4) + r(C6 - C7)] - ¼[r(C4 - C5) + r(C5 - C6) + r(C2 - C3) + r(C3 - C7)]$, a zero-sum coordinate that vanishes in the aromatic S_0 form and grows as the meso bonds contract and the fusion bonds elongate upon quinoidal distortion; q_2 represents the meso–phenyl stretching mode, defined as the symmetric average of the two anthracene–phenyl bonds, $q_2 = ½[r(C5 - C23) + r(C3 - C34)]$, which shortens as inter-ring conjugation extends in the excited state.

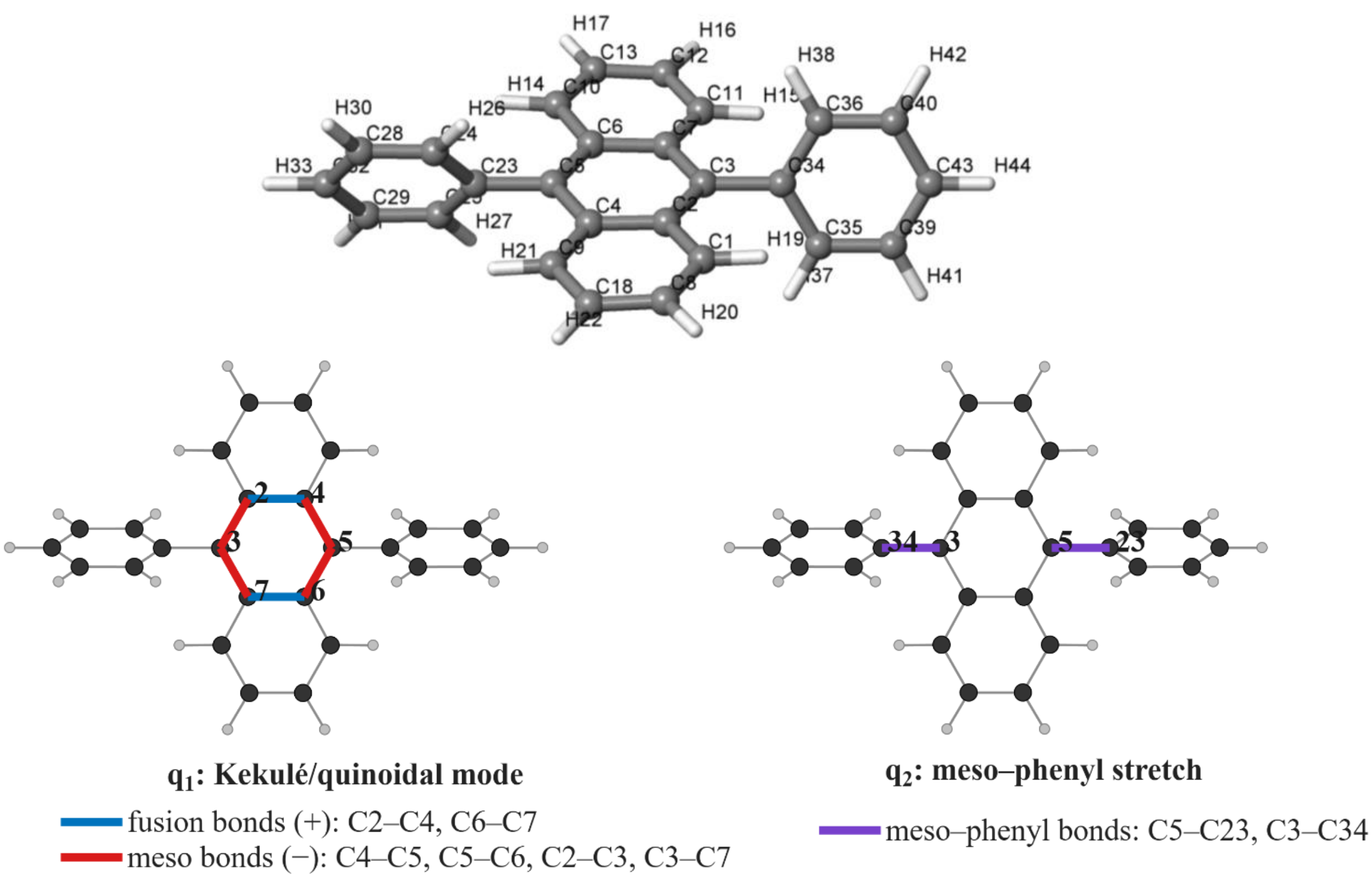

Figure S3. Atom numbering of DPA used throughout this work, corresponding to the standard orientation of the T_1-optimized geometry.

2. Stagewise Data of the Optimal-Force Resonance Procedure

Tables S1–S7 summarize the successive stages of the iterative optimal-force procedure driving the S_1 and T_1 states toward resonance, computed at the TDA-M06-2X/6-31+G(d,p) level for the different systems and routes investigated in this work.

Table S1. Ground-state (S_0), lowest-lying singlet (S_1), and lowest-lying triplet (T_1) energies and associated energy differences obtained at each optimization step for 1,4-bis(phenylethynyl)benzene (BPEB) along route 1 (t_1). Electronic-state energies (E_{S_0} , E_{S_1} , E_{T_1}) are reported in atomic units (a.u.), energy differences (ΔE_{S_0} , ΔE_{S_1} , ΔE_{res}) in kcal mol^{-1}, and the magnitude of the external force ($|F_{ext}|$), in nN.

Steps	E_{S_0}	E_{S_1}	E_{T_1}	ΔE_{S_0}	ΔE_{S_1}	ΔE_{res}	$\|F_{ext}\|$
0	-846.338730	-846.210904	-846.255739	-52.077530	28.134745	-23.942785	-
1	-846.337460	-846.210390	-846.255490	-51.436620	28.297913	-23.138707	0.330817
2	-846.337370	-846.210320	-846.255420	-51.422532	28.304824	-23.117708	0.374124
3	-846.337170	-846.210160	-846.255280	-51.384850	28.317077	-23.067773	0.451905
4	-846.336810	-846.209880	-846.255040	-51.315041	28.336680	-22.978361	0.538673
5	-846.336300	-846.209490	-846.254690	-51.212669	28.364672	-22.847997	0.613591
6	-846.335640	-846.208990	-846.254250	-51.073043	28.402172	-22.670871	0.650635
7	-846.334840	-846.208400	-846.253740	-50.892175	28.449613	-22.442562	0.646700
8	-846.333930	-846.207740	-846.253170	-50.681808	28.504874	-22.176934	0.616729
9	-846.332970	-846.207040	-846.252560	-50.456838	28.563760	-21.893079	0.577248
10	-846.331930	-846.206300	-846.251920	-50.206461	28.628657	-21.577804	0.523568
11	-846.330820	-846.205530	-846.251270	-49.915517	28.704401	-21.211116	0.462485
12	-846.329570	-846.204650	-846.250520	-49.601925	28.783693	-20.818232	0.406810
13	-846.328120	-846.203660	-846.249670	-49.228785	28.872486	-20.356299	0.341561
14	-846.326530	-846.202550	-846.248720	-48.825222	28.970404	-19.854818	0.294435
15	-846.324860	-846.201380	-846.247710	-48.412181	29.072831	-19.339350	0.265801
16	-846.323000	-846.200080	-846.246590	-47.950081	29.187710	-18.762371	0.231379
17	-846.321110	-846.198770	-846.245480	-47.459611	29.306884	-18.152727	0.210582
18	-846.319310	-846.197550	-846.244420	-46.991619	29.414272	-17.577347	0.210472
19	-846.317410	-846.196260	-846.243330	-46.481185	29.541207	-16.939978	0.183001
20	-846.315420	-846.194890	-846.242180	-45.956572	29.677405	-16.279167	0.159584
21	-846.313460	-846.193530	-846.241040	-45.447909	29.809351	-15.638558	0.153783
22	-846.311430	-846.192140	-846.239880	-44.897027	29.955600	-14.941427	0.142305
23	-846.309280	-846.190710	-846.238690	-44.296103	30.110880	-14.185223	0.133710
24	-846.307270	-846.189350	-846.237560	-43.748935	30.250587	-13.498348	0.136374
25	-846.305290	-846.188020	-846.236450	-43.200125	30.389426	-12.810700	0.136049
26	-846.303180	-846.186640	-846.235310	-42.589928	30.544059	-12.045869	0.132083
27	-846.301430	-846.185520	-846.234390	-42.068970	30.667319	-11.401652	0.139984
28	-846.299530	-846.184250	-846.233360	-41.524205	30.813729	-10.710475	0.139225
29	-846.297490	-846.182880	-846.232250	-40.937410	30.977137	-9.960273	0.134505
30	-846.295210	-846.181410	-846.231080	-40.245137	31.165222	-9.079915	0.130794
31	-846.292820	-846.179870	-846.229850	-39.511437	31.364111	-8.147326	0.131735
32	-846.290270	-846.178250	-846.228580	-38.712527	31.580532	-7.131994	0.133595
33	-846.287850	-846.176690	-846.227330	-37.976894	31.774543	-6.202351	0.135406
34	-846.285390	-846.175110	-846.226070	-37.228290	31.972990	-5.255301	0.136482

35	-846.283180	-846.173710	-846.224940	-36.545520	32.147767	-4.397753	0.137847
36	-846.280910	-846.172260	-846.223780	-35.850182	32.324682	-3.525501	0.138474
37	-846.278890	-846.170990	-846.222750	-35.225373	32.479364	-2.746008	0.140430
38	-846.277010	-846.169800	-846.221800	-34.646453	32.629884	-2.016569	0.142295
39	-846.274880	-846.168440	-846.220710	-33.991158	32.798448	-1.192710	0.140423
40	-846.272930	-846.167260	-846.219770	-33.363560	32.949946	-0.413614	0.141438
41	-846.271840	-846.166580	-846.219210	-33.024460	33.027652	0.003192	0.139728

Table S2. Ground-state (S_0), lowest-lying singlet (S_1), and lowest-lying triplet (T_1) energies and associated energy differences obtained at each optimization step for 1,4-bis(phenylethynyl)benzene (BPEB) along route 2 (t_2). Electronic-state energies (E_{S_0} , E_{S_1} , E_{T_1}) are reported in atomic units (a.u.), energy differences (ΔE_{S_0} , ΔE_{S_1} , ΔE_{res}) in kcal mol^{-1}, and the magnitude of the external force ($|F_{ext}|$), in nN.

Steps	E_{S_0}	E_{S_1}	E_{T_1}	ΔE_{S_0}	ΔE_{S_1}	ΔE_{res}	$\lvert F_{ext} \rvert$
0	-846.338730	-846.210904	-846.255739	-52.077530	28.134745	-23.942785	-
1	-846.337450	-846.210390	-846.255490	-51.435047	28.298248	-23.136799	0.331526
2	-846.337350	-846.210310	-846.255420	-51.413346	28.307207	-23.106139	0.379378
3	-846.337110	-846.210130	-846.255270	-51.357798	28.324387	-23.033411	0.470669
4	-846.336680	-846.209820	-846.255000	-51.254055	28.353526	-22.900529	0.587593
5	-846.336040	-846.209360	-846.254620	-51.097128	28.396185	-22.700943	0.716713
6	-846.335190	-846.208760	-846.254110	-50.876513	28.455639	-22.420874	0.840810
7	-846.334070	-846.207990	-846.253470	-50.576617	28.536898	-22.039719	0.950327
8	-846.332720	-846.207070	-846.252710	-50.207872	28.637897	-21.569974	1.048143
9	-846.331200	-846.206040	-846.251860	-49.783804	28.756488	-21.027316	1.128141
10	-846.329540	-846.204950	-846.251000	-49.289276	28.897583	-20.391693	1.178316
11	-846.327620	-846.203670	-846.249980	-48.716024	29.060013	-19.656012	1.203331
12	-846.325500	-846.202290	-846.248890	-48.071337	29.241995	-18.829342	1.214631
13	-846.323160	-846.200780	-846.247700	-47.35234	29.445649	-17.906691	1.210747
14	-846.320610	-846.199120	-846.246410	-46.559409	29.67503	-16.884379	1.192774
15	-846.317960	-846.197400	-846.245070	-45.736286	29.915218	-15.821068	1.167431
16	-846.315300	-846.195680	-846.243730	-44.907265	30.154696	-14.752569	1.146584
17	-846.312710	-846.194010	-846.242440	-44.096637	30.388269	-13.708368	1.129354
18	-846.310170	-846.192380	-846.241170	-43.298494	30.617548	-12.680945	1.099712
19	-846.307700	-846.190800	-846.239940	-42.516909	30.839543	-11.677366	1.068589
20	-846.305200	-846.189200	-846.238700	-41.732216	31.061575	-10.670641	1.029555
21	-846.302770	-846.187630	-846.237470	-40.971342	31.277198	-9.694144	0.992701
22	-846.300420	-846.186120	-846.236290	-40.240351	31.483501	-8.756849	0.959724
23	-846.298100	-846.184640	-846.235140	-39.511014	31.687178	-7.823836	0.923285
24	-846.295700	-846.183110	-846.233940	-38.753729	31.899347	-6.854382	0.882377
25	-846.293320	-846.181580	-846.232740	-38.016831	32.103482	-5.913349	0.848295
26	-846.291030	-846.180100	-846.231570	-37.312318	32.296692	-5.015626	0.819794
27	-846.288920	-846.178750	-846.230500	-36.660573	32.470318	-4.190255	0.779599
28	-846.286860	-846.177410	-846.229400	-36.057604	32.625184	-3.43242	0.730877
29	-846.284950	-846.176140	-846.228340	-35.522534	32.75626	-2.766274	0.676724
30	-846.284110	-846.175570	-846.227890	-35.279392	32.828819	-2.450573	0.720952
31	-846.282460	-846.174510	-846.227010	-34.798465	32.942397	-1.856068	0.923657

32	-846.281000	-846.173530	-846.226160	-34.409893	33.026407	-1.383487	0.759234
33	-846.279610	-846.172560	-846.225280	-34.088825	33.083694	-1.005131	0.620206
34	-846.278270	-846.171600	-846.224390	-33.813658	33.125298	-0.68836	0.495625
35	-846.277010	-846.170660	-846.223480	-33.589555	33.147625	-0.441929	0.394227
36	-846.275840	-846.169760	-846.222590	-33.416554	33.14969	-0.266864	0.321744
37	-846.274800	-846.168930	-846.221740	-33.290863	33.138799	-0.152064	0.263978
38	-846.273700	-846.168060	-846.220830	-33.173987	33.117114	-0.056874	0.187886
39	-846.272670	-846.167220	-846.219940	-33.090158	33.081779	-0.008379	0.146505

Table S3. Ground-state (S_0), lowest-lying singlet (S_1), and lowest-lying triplet (T_1) energies and associated energy differences obtained at each optimization step for 1,4-bis(phenylethynyl)benzene (BPEB) along route 3 (t_3). Electronic-state energies (E_{S_0} , E_{S_1} , E_{T_1}) are reported in atomic units (a.u.), energy differences (ΔE_{S_0} , ΔE_{S_1} , ΔE_{res}) in kcal mol^{-1}, and the magnitude of the external force ($|F_{ext}|$), in nN.

Steps	E_{S_0}	E_{S_1}	E_{T_1}	ΔE_{S_0}	ΔE_{S_1}	ΔE_{res}	$\|F_{ext}\|$
0	-846.338730	-846.210904	-846.255739	-52.077530	28.134745	-23.942785	-
1	-846.337450	-846.210390	-846.255490	-51.434811	28.298299	-23.136512	0.331633
2	-846.337350	-846.210310	-846.255420	-51.411079	28.307745	-23.103333	0.380656
3	-846.337100	-846.210120	-846.255260	-51.350967	28.325926	-23.025042	0.474028
4	-846.336650	-846.209800	-846.254990	-51.239150	28.356813	-22.882337	0.599502
5	-846.335980	-846.209330	-846.254590	-51.068217	28.403232	-22.664986	0.744207
6	-846.335050	-846.208690	-846.254060	-50.822064	28.469680	-22.352384	0.893912
7	-846.333820	-846.207850	-846.253370	-50.479143	28.562883	-21.916260	1.043122
8	-846.332270	-846.206810	-846.252520	-50.042106	28.683970	-21.358137	1.196343
9	-846.330450	-846.205600	-846.251550	-49.514171	28.833197	-20.680974	1.344651
10	-846.328360	-846.204240	-846.250480	-48.866886	29.019077	-19.847809	1.471898
11	-846.325830	-846.202590	-846.249200	-48.085439	29.244954	-18.840485	1.582837
12	-846.322930	-846.200740	-846.247770	-47.167303	29.510616	-17.656686	1.680736
13	-846.319690	-846.198680	-846.246190	-46.116368	29.818621	-16.297747	1.762464
14	-846.316110	-846.196420	-846.244500	-44.930263	30.176464	-14.753800	1.807841
15	-846.312390	-846.194090	-846.242770	-43.691291	30.547639	-13.143652	1.814466
16	-846.308960	-846.191950	-846.241180	-42.530699	30.893101	-11.637598	1.816605
17	-846.305760	-846.189970	-846.239730	-41.437557	31.219897	-10.217661	1.780755
18	-846.302760	-846.188120	-846.238360	-40.411826	31.525046	-8.886780	1.710337
19	-846.300020	-846.186420	-846.237090	-39.486762	31.797164	-7.689598	1.630689
20	-846.297490	-846.184840	-846.235910	-38.643496	32.043293	-6.600203	1.542501
21	-846.295200	-846.183410	-846.234820	-37.887369	32.259091	-5.628278	1.443120
22	-846.293080	-846.182060	-846.233760	-37.222278	32.445064	-4.777214	1.342811
23	-846.291180	-846.180820	-846.232770	-36.649023	32.599424	-4.049599	1.251590
24	-846.289400	-846.179650	-846.231820	-36.132170	32.734326	-3.397843	1.154590
25	-846.287720	-846.178540	-846.230900	-35.655620	32.854574	-2.801046	1.053190
26	-846.286160	-846.177480	-846.230000	-35.243896	32.953197	-2.290699	0.964052
27	-846.284720	-846.176490	-846.229120	-34.888171	33.031656	-1.856515	0.879315
28	-846.283350	-846.175520	-846.228260	-34.570134	33.095396	-1.474738	0.794945
29	-846.282050	-846.174570	-846.227390	-34.299582	33.141260	-1.158322	0.716742
30	-846.280930	-846.173800	-846.226720	-34.019144	33.208131	-0.811013	0.757563

31	-846.280040	-846.173180	-846.226140	-33.820901	33.236796	-0.584105	0.833919
32	-846.279050	-846.172420	-846.225400	-33.662478	33.245330	-0.417149	0.677148
33	-846.278040	-846.171630	-846.224610	-33.528344	33.242841	-0.285502	0.547572
34	-846.277020	-846.170820	-846.223770	-33.412777	33.230045	-0.182733	0.434348
35	-846.276020	-846.170000	-846.222930	-33.315599	33.209813	-0.105786	0.344451
36	-846.275110	-846.169250	-846.222130	-33.246080	33.181442	-0.064638	0.293072
37	-846.274100	-846.168410	-846.221230	-33.175372	33.146029	-0.029343	0.214397
38	-846.273070	-846.167550	-846.220300	-33.110669	33.102549	-0.008121	0.157870
39	-846.272330	-846.166960	-846.219650	-33.056064	33.063189	0.007125	0.140860

Table S4. Ground-state (S_0), lowest-lying singlet (S_1), and lowest-lying triplet (T_1) energies and associated energy differences obtained at each optimization step for Anthracene along route 1 (t_1). Electronic-state energies (E_{S_0} , E_{S_1} , E_{T_1}) are reported in atomic units (a.u.), energy differences (ΔE_{S_0}, ΔE_{S_1}, ΔE_{res}) in kcal mol^{-1}, and the magnitude of the external force ($|F_{ext}|$), in nN.

Steps	E_{S_0}	E_{S_1}	E_{T_1}	ΔE_{S_0}	ΔE_{S_1}	ΔE_{res}	$\lvert F_{ext}\rvert$
0	-539.308158	-539.184960	-539.243136	-40.801795	36.505882	-4.295913	-
1	-539.306720	-539.184540	-539.243000	-39.980608	36.685028	-3.295580	0.471224
2	-539.304610	-539.183710	-539.242480	-38.981370	36.879649	-2.101721	1.003707
3	-539.302700	-539.182710	-539.241830	-38.196584	37.096675	-1.099908	1.426015
4	-539.300470	-539.181450	-539.240880	-37.391852	37.293248	-0.098604	1.846523
5	-539.300200	-539.181270	-539.240740	-37.315467	37.315982	0.000515	1.882560

Table S5. Ground-state (S_0), lowest-lying singlet (S_1), and lowest-lying triplet (T_1) energies and associated energy differences obtained at each optimization step for Anthracene along route 2 (t_2). Electronic-state energies (E_{S_0} , E_{S_1} , E_{T_1}) are reported in atomic units (a.u.), energy differences (ΔE_{S_0}, ΔE_{S_1}, ΔE_{res}) in kcal mol^{-1}, and the magnitude of the external force ($|F_{ext}|$), in nN.

Steps	E_{S_0}	E_{S_1}	E_{T_1}	ΔE_{S_0}	ΔE_{S_1}	ΔE_{res}	$\lvert F_{ext}\rvert$
0	-539.308158	-539.184960	-539.243136	-40.801795	36.505882	-4.295913	-
1	-539.300280	-539.181420	-539.240850	-37.290038	37.295809	0.005771	1.962615

Table S6. Ground-state (S_0), lowest-lying singlet (S_1), and lowest-lying triplet (T_1) energies and associated energy differences obtained at each optimization step for 9,10-diphenylanthracene (DPA) along route 1 (t_1). Electronic-state energies (E_{S_0} , E_{S_1} , E_{T_1}) are reported in atomic units (a.u.), energy differences (ΔE_{S_0}, ΔE_{S_1}, ΔE_{res}) in kcal mol^{-1}, and the magnitude of the external force ($|F_{ext}|$), in nN.

Steps	E_{S_0}	E_{S_1}	E_{T_1}	ΔE_{S_0}	ΔE_{S_1}	ΔE_{res}	$\lvert F_{ext}\rvert$
0	-1001.246900	-1001.131800	-1001.185900	-38.28952	33.933654	-4.355866	-
1	-1001.247000	-1001.131100	-1001.185600	-38.50389	34.239451	-4.264438	0.035235
2	-1001.246300	-1001.130000	-1001.185100	-38.36128	34.593793	-3.767489	0.346345
3	-1001.245600	-1001.129500	-1001.184800	-38.15081	34.722533	-3.428276	0.759203
4	-1001.245000	-1001.129100	-1001.184500	-37.96612	34.766973	-3.199148	0.801787
5	-1001.244100	-1001.128500	-1001.184000	-37.73070	34.811765	-2.918933	0.764092
6	-1001.243000	-1001.127800	-1001.183400	-37.42728	34.861363	-2.565916	0.683366
7	-1001.241700	-1001.127000	-1001.182600	-37.07272	34.89939	-2.173327	0.590299
8	-1001.240300	-1001.126200	-1001.181800	-36.68388	34.907799	-1.776083	0.521315
9	-1001.238700	-1001.125400	-1001.181000	-36.23272	34.902333	-1.330388	0.464449
10	-1001.237000	-1001.124400	-1001.180100	-35.70597	34.898336	-0.807629	0.402735

11	-1001.235200	-1001.123600	-1001.179200	-35.14818	34.834054	-0.314125	0.325604
12	-1001.233700	-1001.123400	-1001.178500	-34.64677	34.615317	-0.031451	0.184577

Table S7. Ground-state (S_0), lowest-lying singlet (S_1), and lowest-lying triplet (T_1) energies and associated energy differences obtained at each optimization step for 9,10-diphenylanthracene (DPA) along route 2 (t_2). Electronic-state energies (E_{S_0} , E_{S_1} , E_{T_1}) are reported in atomic units (a.u.), energy differences (ΔE_{S_0}, ΔE_{S_1}, ΔE_{res}) in kcal mol^{-1}, and the magnitude of the external force ($|F_{ext}|$), in nN.

Steps	E_{S_0}	E_{S_1}	E_{T_1}	ΔE_{S_0}	ΔE_{S_1}	ΔE_{res}	$\lvert F_{ext}\rvert$
0	-1001.246900	-1001.131800	-1001.185900	-38.289520	33.933654	-4.355866	-
1	-1001.247000	-1001.131100	-1001.185600	-38.504247	34.239338	-4.264909	0.035233
2	-1001.246100	-1001.129800	-1001.184900	-38.416534	34.562424	-3.854110	0.639182
3	-1001.245100	-1001.129200	-1001.184500	-38.047590	34.721667	-3.325923	1.180388
4	-1001.244200	-1001.128600	-1001.184000	-37.773193	34.783458	-2.989735	1.248887
5	-1001.243000	-1001.127800	-1001.183400	-37.428935	34.855445	-2.573490	1.232899
6	-1001.241600	-1001.126900	-1001.182600	-37.001181	34.943152	-2.058029	1.158718
7	-1001.240000	-1001.125900	-1001.181700	-36.536981	35.032008	-1.504974	1.026287
8	-1001.238500	-1001.125000	-1001.180900	-36.112773	35.098706	-1.014067	0.863743
9	-1001.237100	-1001.124200	-1001.180200	-35.740083	35.128123	-0.611959	0.689646
10	-1001.235900	-1001.123500	-1001.179500	-35.400745	35.111168	-0.289577	0.494197
11	-1001.234700	-1001.123100	-1001.178800	-35.081273	34.990385	-0.090888	0.312300
12	-1001.233700	-1001.123100	-1001.178400	-34.703287	34.692714	-0.010574	0.180427

3. Two-Dimensional Resonance Trajectories

Figures S4–S6 show the two-dimensional trajectories of BPEB, anthracene, and DPA along the successive stages of their respective resonance routes. For BPEB (Figure S4), three routes are shown: t_1 (target energy = +1 kcal/mol), t_2 (+6 kcal/mol), and t_3 (0 kcal/mol, full resonance). For anthracene (Figure S5) and DPA (Figure S6), two routes are shown: t_1 (+1 kcal/mol) and t_2 (0 kcal/mol, resonance).

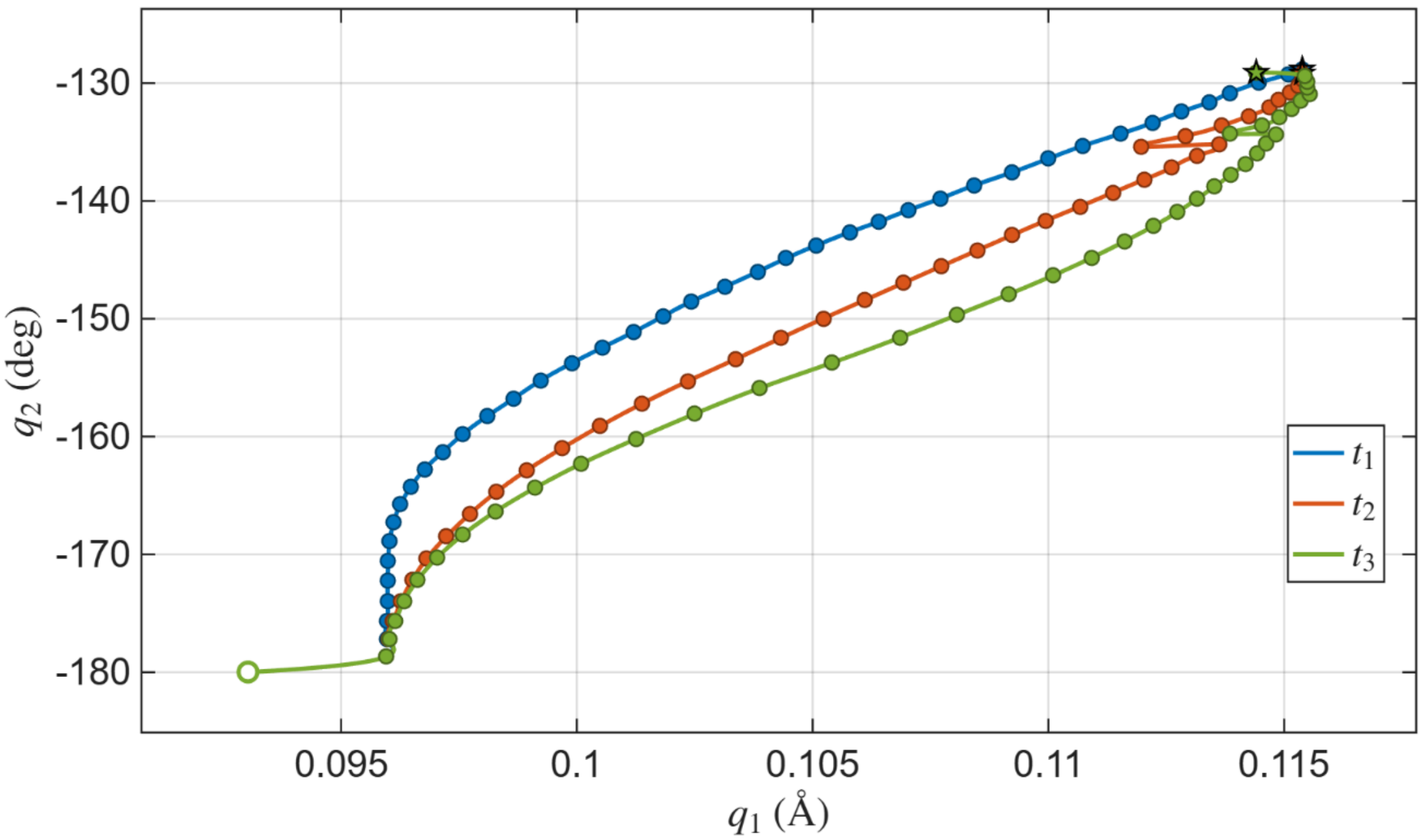

Figure S4. Two-dimensional trajectories of BPEB along the successive stages of the three resonance routes: t_1 (target energy = +1 kcal/mol), t_2 (+6 kcal/mol) and t_3 (0 kcal/mol, full resonance).

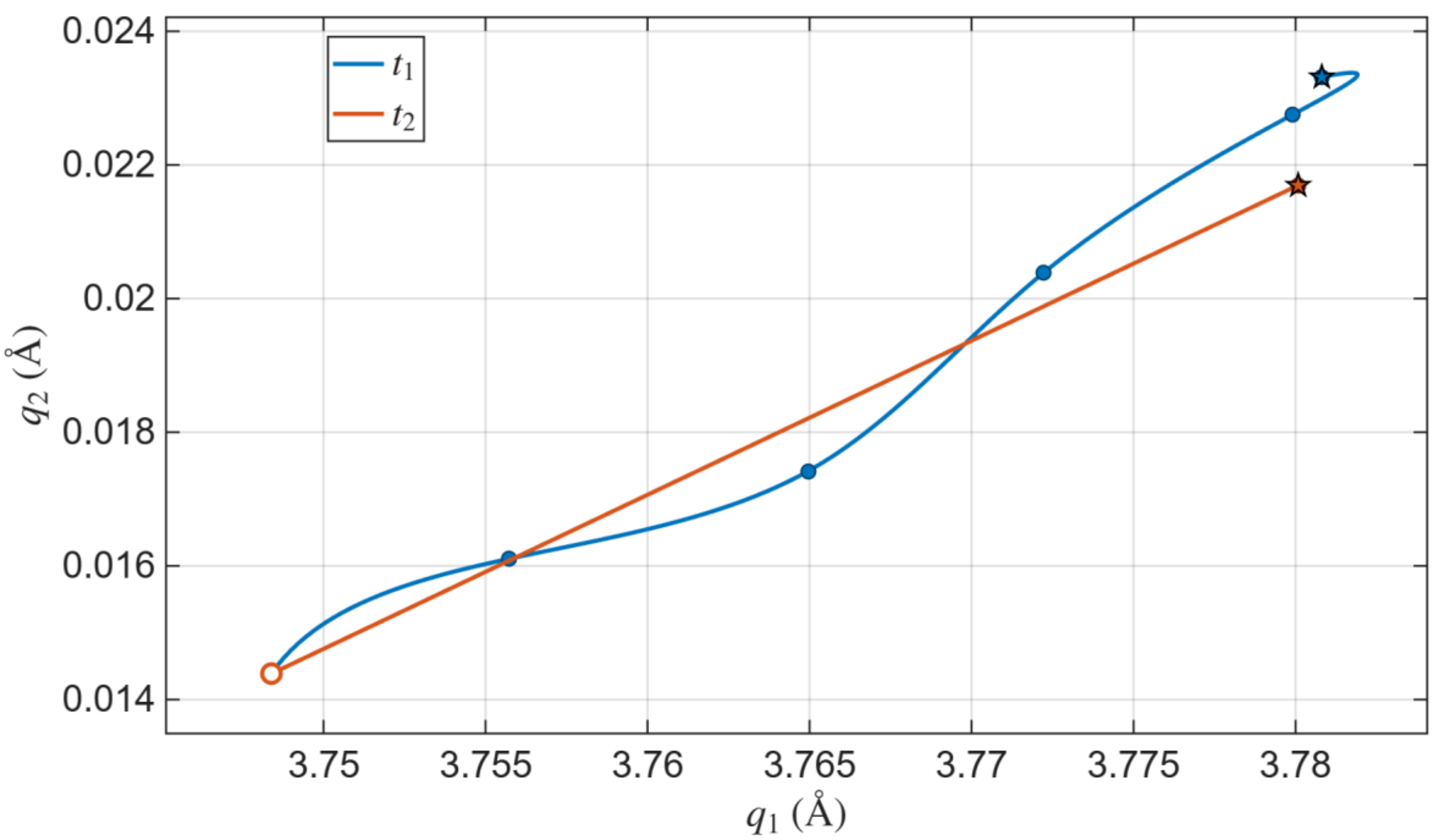

Figure S5. Two-dimensional trajectories of Anthracene along the successive stages of the three resonance routes: t_1 (target energy = +1 kcal/mol), and t_2 (0 kcal/mol, full resonance).

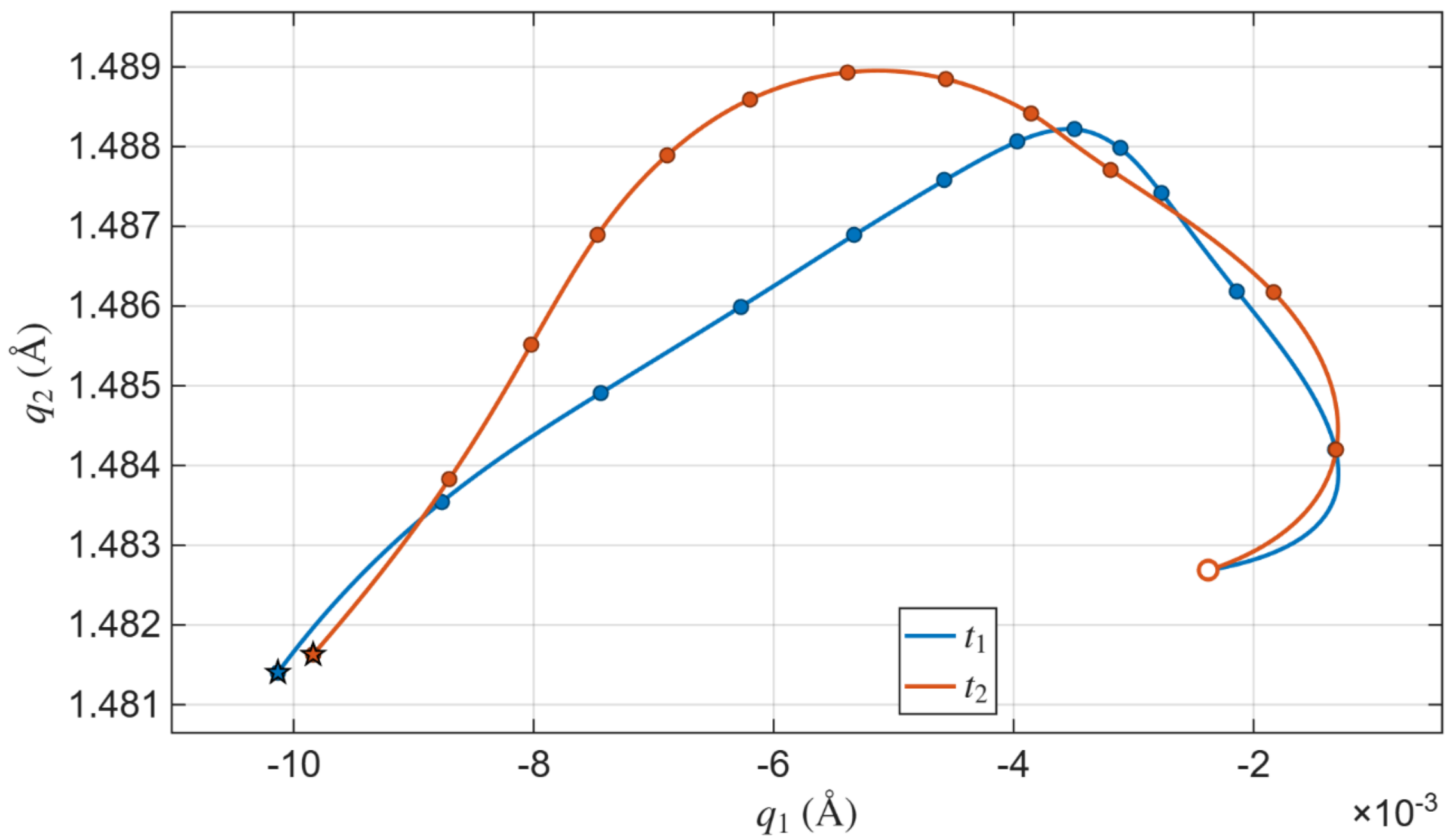

Figure S6. Two-dimensional trajectories of DPA along the successive stages of the three resonance routes: t_1 (target energy = +1 kcal/mol), and t_2 (0 kcal/mol, full resonance).

4. Hole–Electron Descriptors

The electronic character of the T_1 and S_1 states was examined through natural transition orbital (NTO) and hole–electron analyses. The leading NTO weight, $\boldsymbol{\lambda_1}$, quantifies the contribution of the dominant hole–electron NTO pair; values close to unity indicate that the excitation can be described predominantly by a single orbital pair. The excitation energy indicates the energy of the analyzed electronic state at the fixed molecular geometry used in the calculation. The S_r index measures the spatial overlap between the hole and electron distributions, with larger values indicating stronger overlap. The *D* index corresponds to the distance between their spatial centroids and therefore measures the net displacement of the electron relative to the hole. Finally, the *t* index considers both the centroid separation and the spatial extent of the two distributions; negative values indicate substantial hole–electron overlap and the absence of effective spatial charge separation [S1].

Table S8 summarizes the T_1-state descriptors evaluated at the corresponding optimized T_1 geometries of anthracene, BPEB, and DPA. For Table S9, the S_1 state was calculated as a vertical $\boldsymbol{S_0} \rightarrow \boldsymbol{S_1}$excitation at each T_1-optimized geometry. These calculations were performed within the Tamm–Dancoff approximation (TDA) to TDDFT using the M06-2X functional and the 6-31+G(d,p) basis set. The canonical molecular-orbital expansions are consistent with the NTO analysis. At the corresponding T_1-optimized geometries, the T_1 states of anthracene, BPEB, and DPA are dominated by the HOMO (MO 47)→LUMO (MO 48), HOMO (MO 73)→LUMO (MO 74), and HOMO (MO 87)→LUMO (MO 88) configurations, with contributions of 95.60%, 91.00%, and 95.50%, respectively. The vertically calculated S_1 states are dominated by the corresponding HOMO→LUMO configurations, with contributions of 93.84%, 93.07%, and 94.33% for anthracene, BPEB, and DPA, respectively. Minor HOMO−1→LUMO+1 contributions are found for the S_1 states of anthracene (2.64%) and BPEB (2.93%), as well as for the T_1 state of BPEB (2.90%). For both T_1 and S_1, the large $\boldsymbol{\lambda_1}$values show that the excitations are predominantly described by a single NTO pair. The relatively high $\boldsymbol{S_r}$values, coincident hole and electron centroids ($\boldsymbol{D = 0.000}$ Å), and negative *t* indices consistently indicate strongly overlapping hole and electron distributions without significant spatial charge separation. Therefore, the analyzed states are predominantly characterized as locally excited $\boldsymbol{\pi\pi^*}$states rather than long-range charge-transfer states.

Visual inspection of the $\boldsymbol{T_1}$and $\boldsymbol{S_1}$NTOs (Figures S7 and S8) supports the quantitative descriptors. In anthracene, the NTOs are distributed over the fused rings; in BPEB, along the $\boldsymbol{\pi}$-conjugated framework; and in DPA, mainly over the anthracene core. In all cases, the substantial overlap between the hole and electron NTOs is consistent with $\boldsymbol{\pi\pi^*}$excitations without appreciable spatial separation.

4.1. Hole–Electron Descriptors in T_1

Table S8. Hole–electron descriptors of the T_1 states of anthracene, BPEB, and DPA, evaluated at their corresponding optimized T_1 geometries. ΔE denotes the vertical $S_0 \rightarrow T_1$ excitation energy. Energies are given in eV and *D* and *t* in Å; all other quantities are dimensionless (*f*: oscillator factor).

Descriptor	Anthracene	BPEB	DPA
λ_1	0.966	0.940	0.970
ΔE	1.769	2.258	1.660
f	0.000	0.000	0.000
Sr	0.902	0.867	0.896
D	0.000	0.000	0.000

Descriptor	Anthracene	BPEB	DPA
t	−0.967	−0.828	−1.868

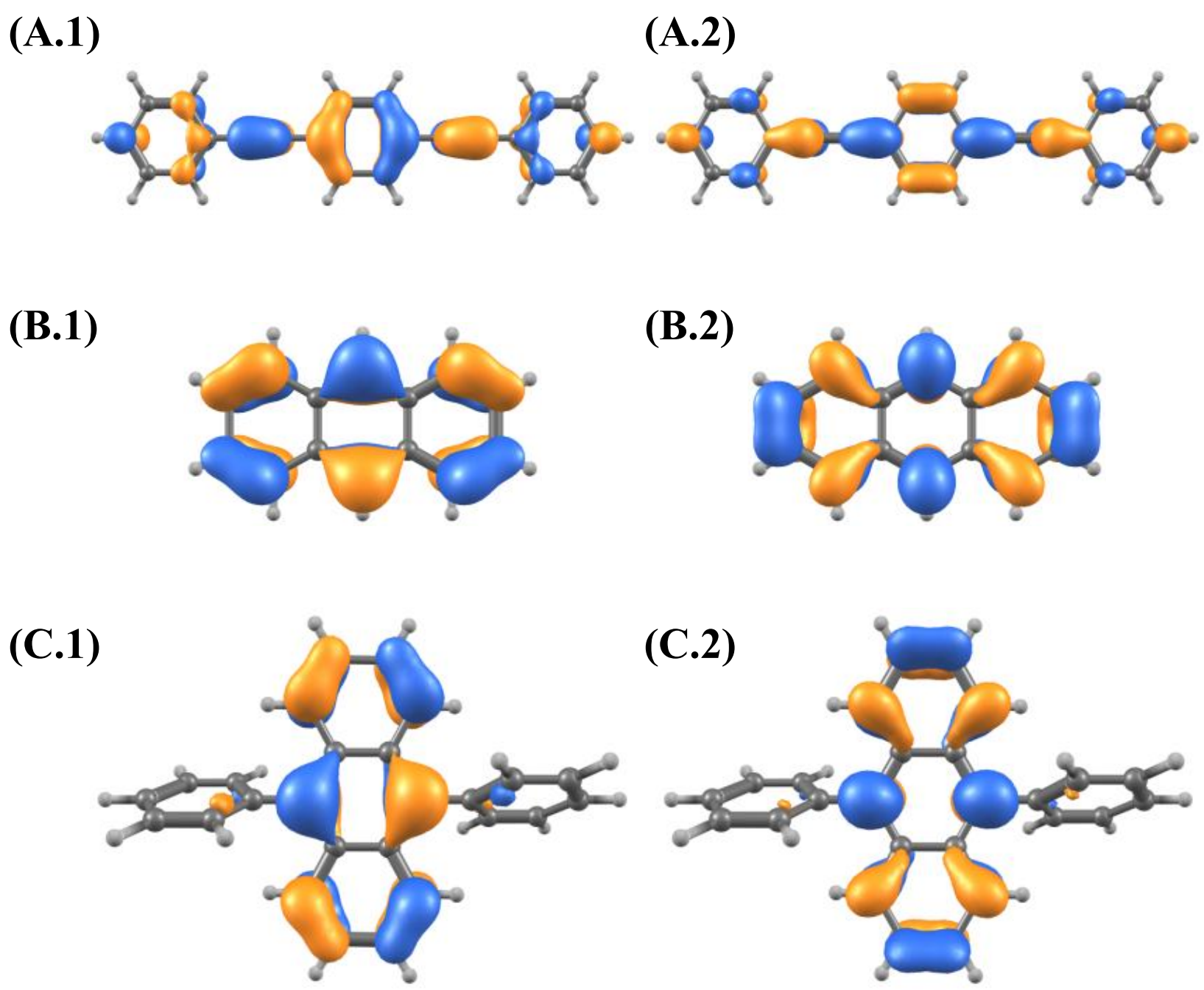

Figure S7. Leading hole–electron natural transition orbital (NTO) pairs for the T_1 states of (A) BPEB, (B) anthracene, and (C) DPA, evaluated at their corresponding optimized T_1 geometries. Panels A.1, B.1, and C.1 show the hole NTOs, whereas panels A.2, B.2, and C.2 show the corresponding electron NTOs.

4.2. Hole–Electron Descriptors in S_1

Table S9. Hole–electron descriptors of the S_1 states of anthracene, BPEB, and DPA, evaluated at their corresponding optimized T_1 geometries. ΔE denotes the vertical $S_0 \rightarrow S_1$ excitation energy. Energies are given in eV and *D* and *t* in Å; all other quantities are dimensionless (*f*: oscillator factor).

Descriptor	Anthracene	BPEB	DPA
λ_1	0.938	0.931	0.943
ΔE	3.352	3.478	3.132
f	0.144	2.674	0.355
Sr	0.880	0.807	0.835
D	0.000	0.000	0.000
t	−0.860	−0.862	−1.780

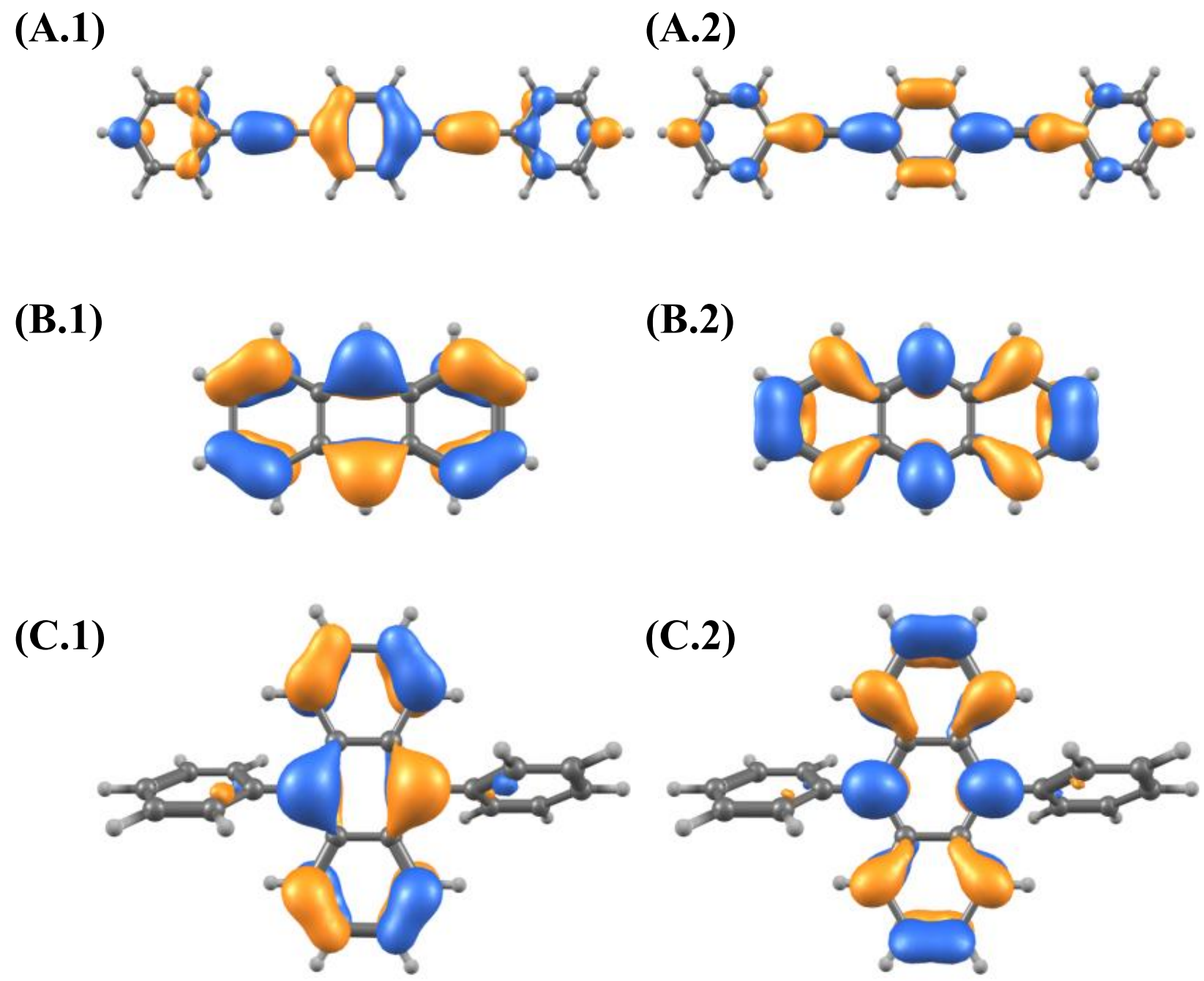

Figure S8. Leading hole–electron natural transition orbital (NTO) pairs for the S_1 states of (A) BPEB, (B) anthracene, and (C) DPA, evaluated at their corresponding optimized T_1 geometries. Panels A.1, B.1, and C.1 show the hole NTOs, whereas panels A.2, B.2, and C.2 show the corresponding electron NTOs.

5. Optimized Cartesian Coordinates

Tables S10–S18 list the Cartesian coordinates (Å, standard orientation) of the S_0, T_1, and S_1 optimized geometries of BPEB (Tables S10–S12), anthracene (Tables S13–S15), and DPA (Tables S16–S18), computed at the TDA-M06-2X/6-31+G(d,p) level.

Table S10. Cartesian coordinates (Å, standard orientation) of BPEB optimized in its S_0 state at the TDA-M06-2X/6-31+G(d,p) level.

6	1.405553000	-0.000002000	0.000000000
6	0.693329000	1.210435000	0.000185000
6	0.693329000	-1.210438000	-0.000185000
1	1.240849000	2.146954000	0.000208000
1	1.240849000	-2.146958000	-0.000209000
6	-0.693329000	1.210435000	-0.000177000
6	-0.693329000	-1.210438000	0.000177000
1	-1.240849000	2.146955000	-0.000194000
1	-1.240849000	-2.146958000	0.000194000
6	-1.405553000	-0.000002000	0.000000000
6	-2.836139000	-0.000002000	0.000000000
6	2.836139000	-0.000002000	0.000000000
6	4.049319000	-0.000001000	0.000000000
6	-5.481654000	0.000000000	0.000000000
6	-6.190561000	1.209182000	-0.073887000
6	-6.190563000	-1.209180000	0.073887000
1	-5.640184000	2.142495000	-0.131377000
1	-5.640188000	-2.142494000	0.131377000
6	-7.581226000	1.204869000	-0.073766000
6	-7.581228000	-1.204866000	0.073766000
1	-8.121370000	2.144492000	-0.131613000
1	-8.121374000	-2.144488000	0.131614000
6	-8.280162000	0.000002000	0.000000000
1	-9.365470000	0.000003000	0.000000000
6	8.280162000	0.000002000	0.000000000
6	7.581226000	1.204869000	0.073762000
6	7.581228000	-1.204866000	-0.073762000
1	8.121370000	2.144492000	0.131607000
1	8.121374000	-2.144488000	-0.131606000
6	6.190561000	1.209182000	0.073883000
6	6.190563000	-1.209181000	-0.073883000
1	5.640184000	2.142495000	0.131369000
1	5.640188000	-2.142495000	-0.131369000
6	5.481654000	0.000000000	0.000000000
1	9.365470000	0.000003000	0.000000000
6	-4.049319000	-0.000001000	0.000000000

Table S11. Cartesian coordinates (Å, standard orientation) of BPEB optimized in its T_1 state at the TDA-M06-2X/6-31+G(d,p) level.

6	-1.425228000	-0.000005000	-0.000022000
6	-0.678366000	-1.242582000	0.000007000
6	-0.678371000	1.242574000	-0.000053000
1	-1.232739000	-2.175101000	0.000032000
1	-1.232748000	2.175091000	-0.000074000

6	0.678372000	-1.242579000	0.000021000
6	0.678367000	1.242577000	-0.000039000
1	1.232748000	-2.175097000	0.000056000
1	1.232740000	2.175096000	-0.000051000
6	1.425228000	0.000000000	-0.000016000
6	2.801916000	0.000004000	-0.000024000
6	-2.801916000	-0.000007000	-0.000024000
6	-4.038861000	-0.000010000	-0.000067000
6	5.444165000	0.000002000	-0.000009000
6	6.164848000	-1.218597000	0.000080000
6	6.164851000	1.218600000	-0.000074000
1	5.615127000	-2.153905000	0.000130000
1	5.615132000	2.153909000	-0.000144000
6	7.550684000	-1.210069000	0.000103000
6	7.550688000	1.210070000	-0.000056000
1	8.092719000	-2.150426000	0.000172000
1	8.092725000	2.150425000	-0.000110000
6	8.251881000	-0.000001000	0.000034000
1	9.336878000	-0.000002000	0.000051000
6	-8.251881000	0.000008000	0.000072000
6	-7.550691000	-1.210064000	0.000125000
6	-7.550681000	1.210075000	-0.000033000
1	-8.092731000	-2.150418000	0.000204000
1	-8.092713000	2.150433000	-0.000078000
6	-6.164855000	-1.218599000	0.000075000
6	-6.164844000	1.218598000	-0.000080000
1	-5.615139000	-2.153910000	0.000113000
1	-5.615121000	2.153904000	-0.000162000
6	-5.444165000	-0.000003000	-0.000025000
1	-9.336878000	0.000013000	0.000111000
6	4.038861000	0.000005000	-0.000039000

Table S12. Cartesian coordinates (Å, standard orientation) of BPEB optimized in its S_1 state at the TDA-M06-2X/6-31+G(d,p) level.

6	-1.417999000	0.000087000	0.000139000
6	-0.684379000	-1.231037000	0.000186000
6	-0.684351000	1.231193000	0.000111000
1	-1.236641000	-2.164806000	0.000215000
1	-1.236591000	2.164976000	0.000077000
6	0.684351000	-1.231053000	0.000201000
6	0.684380000	1.231177000	0.000137000
1	1.236591000	-2.164835000	0.000233000
1	1.236641000	2.164947000	0.000115000
6	1.417999000	0.000054000	0.000161000
6	2.807662000	0.000033000	0.000109000
6	-2.807662000	0.000101000	0.000128000
6	-4.042814000	0.000089000	0.000126000
6	5.443625000	-0.000014000	-0.000030000
6	6.167966000	-1.221843000	-0.000115000
6	6.168020000	1.221783000	-0.000020000
1	5.618457000	-2.157478000	-0.000123000

1	5.618553000	2.157442000	0.000047000
6	7.552443000	-1.211602000	-0.000197000
6	7.552497000	1.211481000	-0.000100000
1	8.095423000	-2.151600000	-0.000268000
1	8.095518000	2.151455000	-0.000095000
6	8.254596000	-0.000076000	-0.000190000
1	9.339798000	-0.000100000	-0.000256000
6	-8.254596000	-0.000125000	-0.000194000
6	-7.552409000	-1.211631000	-0.000130000
6	-7.552532000	1.211452000	-0.000155000
1	-8.095361000	-2.151645000	-0.000154000
1	-8.095580000	2.151411000	-0.000200000
6	-6.167931000	-1.221832000	-0.000030000
6	-6.168055000	1.221794000	-0.000059000
1	-5.618395000	-2.157451000	0.000026000
1	-5.618614000	2.157469000	-0.000025000
6	-5.443625000	0.000018000	0.000007000
1	-9.339797000	-0.000180000	-0.000269000
6	4.042814000	0.000016000	0.000027000

Table S13. Cartesian coordinates (Å, standard orientation) of Anthracene optimized in its S_0 state at the TDA-M06-2X/6-31+G(d,p) level.

6	0.000000000	2.475782000	-1.405634000
6	0.000000000	1.219632000	-0.718329000
6	0.000000000	0.000000000	-1.400719000
6	0.000000000	1.219632000	0.718329000
6	0.000000000	0.000000000	1.400719000
6	0.000000000	-1.219632000	0.718329000
6	0.000000000	-1.219632000	-0.718329000
6	0.000000000	3.652389000	-0.714386000
6	0.000000000	2.475782000	1.405634000
1	0.000000000	0.000000000	2.488884000
6	0.000000000	-2.475782000	1.405634000
6	0.000000000	-2.475782000	-1.405634000
6	0.000000000	-3.652389000	-0.714386000
6	0.000000000	-3.652389000	0.714386000
1	0.000000000	-2.471959000	2.492403000
1	0.000000000	-2.471959000	-2.492403000
1	0.000000000	-4.597664000	-1.247697000
1	0.000000000	-4.597664000	1.247697000
6	0.000000000	3.652389000	0.714386000
1	0.000000000	2.471959000	-2.492403000
1	0.000000000	0.000000000	-2.488884000
1	0.000000000	4.597664000	-1.247697000
1	0.000000000	2.471959000	2.492403000
1	0.000000000	4.597664000	1.247697000

Table S14. Cartesian coordinates (Å, standard orientation) of Anthracene optimized in its T_1 state at the TDA-M06-2X/6-31+G(d,p) level.

6	0.000000000	2.473145000	1.394570000
6	0.000000000	1.248936000	0.717967000
6	0.000000000	0.000000000	1.404054000
6	0.000000000	1.248936000	-0.717967000
6	0.000000000	0.000000000	-1.404054000
6	0.000000000	-1.248936000	-0.717967000
6	0.000000000	-1.248936000	0.717967000
6	0.000000000	3.695880000	0.691231000
6	0.000000000	2.473145000	-1.394570000
1	0.000000000	0.000000000	-2.491000000
6	0.000000000	-2.473145000	-1.394570000
6	0.000000000	-2.473145000	1.394570000
6	0.000000000	-3.695880000	0.691231000
6	0.000000000	-3.695880000	-0.691231000
1	0.000000000	-2.474379000	-2.481077000
1	0.000000000	-2.474379000	2.481077000
1	0.000000000	-4.631512000	1.240557000
1	0.000000000	-4.631512000	-1.240557000
6	0.000000000	3.695880000	-0.691231000
1	0.000000000	2.474379000	2.481077000
1	0.000000000	0.000000000	2.491000000
1	0.000000000	4.631512000	1.240557000
1	0.000000000	2.474379000	-2.481077000
1	0.000000000	4.631512000	-1.240557000

Table S15. Cartesian coordinates (Å, standard orientation) of Anthracene optimized in its S_1 state at the TDA-M06-2X/6-31+G(d,p) level.

6	0.000000000	2.473035000	1.398716000
6	0.000000000	1.239825000	0.720857000
6	0.000000000	0.000000000	1.396634000
6	0.000000000	1.239825000	-0.720857000
6	0.000000000	0.000000000	-1.396634000
6	0.000000000	-1.239825000	-0.720857000
6	0.000000000	-1.239825000	0.720857000
6	0.000000000	3.690026000	0.695973000
6	0.000000000	2.473035000	-1.398716000
1	0.000000000	0.000000000	-2.485008000
6	0.000000000	-2.473035000	-1.398716000
6	0.000000000	-2.473035000	1.398716000
6	0.000000000	-3.690026000	0.695973000
6	0.000000000	-3.690026000	-0.695973000
1	0.000000000	-2.474876000	-2.485250000
1	0.000000000	-2.474876000	2.485250000
1	0.000000000	-4.626888000	1.242889000
1	0.000000000	-4.626888000	-1.242889000
6	0.000000000	3.690026000	-0.695973000
1	0.000000000	2.474876000	2.485250000
1	0.000000000	0.000000000	2.485008000
1	0.000000000	4.626888000	1.242889000

1	0.000000000	2.474876000	-2.485250000
1	0.000000000	4.626888000	-1.242889000

Table S16. Cartesian coordinates (Å, standard orientation) of DPA optimized in its S_0 state at the TDA-M06-2X/6-31+G(d,p) level.

6	1.402362000	-2.481461000	0.030130000
6	0.719013000	-1.219676000	0.005407000
6	1.420704000	0.000005000	0.000002000
6	-0.719007000	-1.219682000	-0.005384000
6	-1.420707000	-0.000003000	0.000003000
6	-0.719015000	1.219680000	0.005386000
6	0.719006000	1.219684000	-0.005407000
6	0.712575000	-3.658701000	0.019804000
6	-1.402341000	-2.481476000	-0.030105000
6	-1.402359000	2.481468000	0.030102000
6	1.402345000	2.481474000	-0.030137000
6	0.712548000	3.658709000	-0.019813000
6	-0.712567000	3.658706000	0.019758000
1	-2.486802000	2.485247000	0.060285000
1	2.486788000	2.485257000	-0.060316000
1	1.248864000	4.602195000	-0.040597000
1	-1.248887000	4.602190000	0.040527000
6	-0.712541000	-3.658708000	-0.019765000
1	2.486806000	-2.485234000	0.060307000
1	1.248898000	-4.602183000	0.040584000
1	-2.486784000	-2.485263000	-0.060288000
1	-1.248853000	-4.602197000	-0.040537000
6	-2.913538000	-0.000005000	0.000002000
6	-3.623400000	-0.256453000	1.177438000
6	-3.623399000	0.256442000	-1.177435000
1	-3.073998000	-0.456814000	2.093432000
1	-3.073997000	0.456805000	-2.093427000
6	-5.017263000	-0.255455000	1.178408000
6	-5.017262000	0.255441000	-1.178406000
1	-5.555962000	-0.454273000	2.099756000
1	-5.555961000	0.454258000	-2.099754000
6	-5.717199000	-0.000008000	0.000001000
1	-6.802677000	-0.000009000	0.000000000
6	2.913535000	0.000005000	0.000002000
6	3.623396000	-0.256452000	-1.177433000
6	3.623397000	0.256458000	1.177438000
1	3.073993000	-0.456819000	-2.093424000
1	3.073994000	0.456825000	2.093430000
6	5.017259000	-0.255453000	-1.178403000
6	5.017259000	0.255458000	1.178408000
1	5.555958000	-0.454276000	-2.099750000
1	5.555959000	0.454281000	2.099754000
6	5.717195000	0.000003000	0.000002000
1	6.802674000	0.000002000	0.000002000

Table S17. Cartesian coordinates (Å, standard orientation) of DPA optimized in its T_1 state at the TDA-M06-2X/6-31+G(d,p) level.

6	1.387607000	2.481913000	-0.070958000
6	0.718125000	1.251687000	-0.014795000
6	1.427739000	-0.000005000	0.000014000
6	-0.718115000	1.251687000	0.014786000
6	-1.427729000	0.000001000	-0.000007000
6	-0.718120000	-1.251688000	-0.014790000
6	0.718119000	-1.251695000	0.014815000
6	0.689147000	3.703759000	-0.042519000
6	-1.387608000	2.481909000	0.070920000
6	-1.387618000	-2.481906000	-0.070957000
6	1.387593000	-2.481925000	0.071006000
6	0.689127000	-3.703766000	0.042549000
6	-0.689172000	-3.703758000	-0.042478000
1	-2.469656000	-2.493444000	-0.139381000
1	2.469628000	-2.493487000	0.139451000
1	1.241656000	-4.636765000	0.082839000
1	-1.241715000	-4.636749000	-0.082744000
6	-0.689155000	3.703758000	0.042451000
1	2.469645000	2.493469000	-0.139368000
1	1.241683000	4.636754000	-0.082790000
1	-2.469648000	2.493454000	0.139309000
1	-1.241696000	4.636752000	0.082690000
6	-2.910414000	0.000003000	-0.000006000
6	-3.625548000	0.488455000	-1.101811000
6	-3.625543000	-0.488446000	1.101805000
1	-3.077672000	0.866430000	-1.960914000
1	-3.077662000	-0.866423000	1.960904000
6	-5.018565000	0.485054000	-1.103664000
6	-5.018560000	-0.485041000	1.103668000
1	-5.556814000	0.860766000	-1.968403000
1	-5.556804000	-0.860751000	1.968409000
6	-5.719212000	0.000008000	0.000004000
1	-6.804648000	0.000011000	0.000008000
6	2.910422000	-0.000005000	0.000008000
6	3.625565000	0.488420000	1.101822000
6	3.625550000	-0.488423000	-1.101821000
1	3.077695000	0.866368000	1.960941000
1	3.077667000	-0.866369000	-1.960932000
6	5.018582000	0.485019000	1.103668000
6	5.018566000	-0.485020000	-1.103688000
1	5.556835000	0.860708000	1.968413000
1	5.556807000	-0.860705000	-1.968443000
6	5.719224000	0.000000000	-0.000016000
1	6.804660000	0.000002000	-0.000024000

Table S18. Cartesian coordinates (Å, standard orientation) of DPA optimized in its S_1 state at the TDA-M06-2X/6-31+G(d,p) level.

6	1.382173000	2.481391000	-0.152998000
6	0.720383000	1.241413000	-0.032717000
6	1.426768000	-0.000071000	0.000010000
6	-0.720285000	1.241493000	0.032726000
6	-1.426789000	0.000055000	-0.000007000
6	-0.720392000	-1.241437000	-0.032759000
6	0.720276000	-1.241505000	0.032702000
6	0.689602000	3.696906000	-0.090116000
6	-1.381887000	2.481566000	0.153114000
6	-1.382135000	-2.481427000	-0.153205000
6	1.381916000	-2.481567000	0.152989000
6	0.689203000	-3.697001000	0.090073000
6	-0.689531000	-3.696931000	-0.090410000
1	-2.455867000	-2.495305000	-0.300606000
1	2.455650000	-2.495556000	0.300366000
1	1.235640000	-4.630510000	0.174974000
1	-1.236048000	-4.630382000	-0.175442000
6	-0.689141000	3.696990000	0.090306000
1	2.455920000	2.495242000	-0.300308000
1	1.236148000	4.630352000	-0.175014000
1	-2.455616000	2.495580000	0.300509000
1	-1.235556000	4.630505000	0.175296000
6	-2.902772000	0.000085000	0.000013000
6	-3.624810000	0.663869000	-1.007496000
6	-3.624786000	-0.663705000	1.007533000
1	-3.079192000	1.169956000	-1.799255000
1	-3.079147000	-1.169819000	1.799262000
6	-5.015979000	0.656872000	-1.011157000
6	-5.015954000	-0.656708000	1.011227000
1	-5.553634000	1.163020000	-1.807141000
1	-5.553589000	-1.162866000	1.807217000
6	-5.718447000	0.000086000	0.000046000
1	-6.803836000	0.000088000	0.000060000
6	2.902751000	-0.000092000	0.000025000
6	3.624754000	0.664056000	1.007309000
6	3.624778000	-0.664207000	-1.007266000
1	3.079110000	1.170459000	1.798849000
1	3.079153000	-1.170588000	-1.798834000
6	5.015924000	0.657093000	1.010984000
6	5.015948000	-0.657193000	-1.010944000
1	5.553561000	1.163546000	1.806785000
1	5.553602000	-1.163611000	-1.806758000
6	5.718416000	-0.000045000	0.000024000
1	6.803804000	-0.000026000	0.000024000

6. Resonance Cartesian Coordinates

Tables S19–S25 list the Cartesian coordinates of the resonance structures obtained at the final stage of each optimal-force route, at the TDA-M06-2X/6-31+G(d,p) level: BPEB along routes t_1–t_3 (Tables S19–S21), anthracene along routes t_1–t_2 (Tables S22–S23), and DPA along routes t_1–t_2 (Tables S24–S25).

Table S19. Cartesian coordinates of the BPEB resonance structure (cycle 41) obtained at the final stage of route 1 (t_1) optimal-force procedure, at the TDA-M06-2X/6-31+G(d,p) level.

6	-1.363865197	0.000458660	2.772233065
6	-0.674385043	-1.252338437	3.088504190
6	-0.674682893	1.249857131	3.102232289
1	-1.250675279	-2.164300166	3.210262322
1	-1.250883334	2.160579167	3.233570182
6	0.674668916	-1.252269988	3.088184920
6	0.674544441	1.249917065	3.101728742
1	1.251151523	-2.164168537	3.209657209
1	1.250681494	2.160698300	3.232661124
6	1.363761442	0.000589823	2.771443727
6	2.375316074	0.003430745	1.837738382
6	-2.375736499	0.003362236	1.838913725
6	-3.066376975	0.003655729	0.816569978
6	3.571634226	0.000675300	-0.506564988
6	3.748884314	-1.216465267	-1.198583173
6	3.791202648	1.215441938	-1.189725780
1	3.577312545	-2.150642145	-0.673851208
1	3.652484390	2.151551418	-0.658358583
6	4.108682299	-1.211218232	-2.539143059
6	4.151157273	1.206543117	-2.530288504
1	4.228745862	-2.152840096	-3.066295520
1	4.304314268	2.146919028	-3.050625397
6	4.305457370	-0.003096113	-3.212436440
1	4.578415215	-0.004246901	-4.262726368
6	-4.304762554	-0.001154303	-3.211801000
6	-4.102731424	-1.209812921	-2.540781210
6	-4.156326667	1.208351822	-2.527099057
1	-4.218131309	-2.151346441	-3.069929715
1	-4.313665651	2.149141117	-3.045638337
6	-3.743516670	-1.215650979	-1.199972188
6	-3.796867740	1.216657151	-1.186289647
1	-3.567775017	-2.150159793	-0.677012220
1	-3.662728766	2.152756740	-0.652965938
6	-3.572024801	0.001432976	-0.505413447
1	-4.577265692	-0.001789987	-4.262289554
6	3.065728726	0.003222062	0.815255783

Table S20. Cartesian coordinates of the BPEB resonance structure (cycle 39) obtained at the final stage of route 2 (t_2) optimal-force procedure, at the TDA-M06-2X/6-31+G(d,p) level.

6	-1.349895962	-0.009321839	2.749248853
6	-0.658500252	-1.257912549	3.076083114
6	-0.667875905	1.244121293	3.077556673

1	-1.232564537	-2.171380486	3.197427358
1	-1.248957501	2.153017428	3.200041652
6	0.690642581	-1.253004764	3.088062333
6	0.681235502	1.249550726	3.089371875
1	1.269393241	-2.162110609	3.219373360
1	1.253038915	2.162907632	3.221677576
6	1.377360043	0.001049571	2.769281188
6	2.390980875	0.005014157	1.838157475
6	-2.363114686	-0.011660405	1.816824617
6	-3.059175486	-0.011181185	0.798520054
6	3.602423185	0.007287727	-0.499486316
6	3.813488198	-1.208872912	-1.183061075
6	3.804549534	1.222676160	-1.186873197
1	3.654706460	-2.143541375	-0.655372969
1	3.638840498	2.157968136	-0.662240398
6	4.191868492	-1.202689281	-2.519126698
6	4.182985819	1.214679714	-2.522949922
1	4.339314841	-2.143872941	-3.039654970
1	4.323489510	2.155149104	-3.046512081
6	4.373008818	0.005543632	-3.196446359
1	4.661728811	0.004777469	-4.243102235
6	-4.419313179	0.005065437	-3.193311306
6	-4.222094261	-1.206439817	-2.526217570
6	-4.217709199	1.210862948	-2.517181111
1	-4.378235185	-2.145039031	-3.048928638
1	-4.370384261	2.153931628	-3.032787217
6	-3.817286494	-1.219284271	-1.197582421
6	-3.813013071	1.212147234	-1.188523403
1	-3.646836404	-2.156591568	-0.678073906
1	-3.639144122	2.144823607	-0.661845652
6	-3.596200126	-0.006520228	-0.511285233
1	-4.729614825	0.009602971	-4.234027924
6	3.084476553	0.007176366	0.817747960

Table S21. Cartesian coordinates of the BPEB resonance structure (cycle 39) obtained at the final stage of route 3 (t_3) optimal-force procedure, at the TDA-M06-2X/6-31+G(d,p) level.

6	-1.385448968	-0.036508546	2.790802683
6	-0.657795877	-1.269934894	3.109427185
6	-0.719264169	1.231586861	3.103410958
1	-1.208023615	-2.195228388	3.252765958
1	-1.314243434	2.129118672	3.242538469
6	0.691278438	-1.237382540	3.066031195
6	0.629654866	1.264956214	3.063027705
1	1.293527279	-2.134004526	3.179280383
1	1.187302040	2.190274670	3.173504399
6	1.342073292	0.029819587	2.733820961
6	2.357242602	0.045949693	1.803683947
6	-2.395784112	-0.053931003	1.856243612
6	-3.082492395	-0.046854661	0.831182307
6	3.593583260	0.019960757	-0.520324051

6	3.806820901	-1.208492094	-1.180540533
6	3.808448546	1.222900896	-1.225189138
1	3.638634886	-2.133541278	-0.638934671
1	3.641773432	2.167485820	-0.717988392
6	4.198317023	-1.226169171	-2.512244915
6	4.200105485	1.191085550	-2.556577388
1	4.346775275	-2.176507397	-3.015377586
1	4.350081304	2.122143791	-3.094172030
6	4.391130723	-0.029995915	-3.207530531
1	4.689494315	-0.049255246	-4.250856798
6	-4.343174434	0.040410234	-3.192158158
6	-4.167110443	-1.183302906	-2.541202046
6	-4.158344664	1.233647432	-2.489405700
1	-4.310626894	-2.112281586	-3.084331218
1	-4.294925716	2.186193643	-2.992265996
6	-3.798290699	-1.220646748	-1.202671292
6	-3.790077858	1.210438013	-1.150386246
1	-3.643189815	-2.167568993	-0.695658851
1	-3.628449332	2.133399475	-0.603048601
6	-3.592383392	-0.020717504	-0.489384090
1	-4.623778814	0.063977858	-4.240934999
6	3.061038835	0.041174705	0.790702948

Table S22. Cartesian coordinates of the Anthracene resonance structure obtained at the final stage of route 1(t_1) optimal-force procedure, at the TDA-M06-2X/6-31+G(d,p) level.

6	-0.001100664	2.507787331	1.384494742
6	-0.003668039	1.272956091	0.717815821
6	-0.005619579	-0.000000764	1.395987333
6	-0.002626653	1.272960169	-0.717823152
6	-0.005483337	0.000000428	-1.395986798
6	-0.003648344	-1.272956931	-0.717814772
6	-0.002680383	-1.272959765	0.717824076
6	0.003774518	3.745005723	0.689588824
6	0.002370384	2.507793895	-1.384500469
1	-0.006408265	-0.000005643	-2.483648743
6	-0.001169156	-2.507787903	-1.384494683
6	0.002399885	-2.507794149	1.384499320
6	0.005972952	-3.745009493	0.689586128
6	0.003720140	-3.745006236	-0.689588816
1	-0.002355358	-2.511257964	-2.471345121
1	0.003878919	-2.511268057	2.471349006
1	0.010209731	-4.675694663	1.247763652
1	0.005960288	-4.675689582	-1.247771905
6	0.005887065	3.745010300	-0.689586816
1	-0.002237666	2.511257710	2.471344407
1	-0.006689384	0.000005229	2.483649150
1	0.006056259	4.675688871	1.247771552
1	0.003823354	2.511267908	-2.471349982
1	0.010030066	4.675695854	-1.247763761

Table S23. Cartesian coordinates of the Anthracene resonance structure obtained at the final stage of route 2 (t_2) optimal-force procedure, at the TDA-M06-2X/6-31+G(d,p) level.

6	-0.000006838	2.506078033	1.383285169
6	-0.000009539	1.271097040	0.717858604
6	-0.000007510	0.000001698	1.396964412
6	-0.000013511	1.271092971	-0.717852487
6	-0.000008515	-0.000001131	-1.396964062
6	0.000021733	-1.271096670	-0.717858421
6	0.000016859	-1.271092442	0.717852320
6	0.000005642	3.744239113	0.689193090
6	-0.000008825	2.506071399	-1.383280457
1	-0.000023347	0.000002285	-2.484648167
6	0.000015196	-2.506078421	-1.383282555
6	0.000014245	-2.506071185	1.383279431
6	-0.000011228	-3.744235116	0.689192505
6	-0.000013379	-3.744239877	-0.689193025
1	0.000012874	-2.509342968	-2.470155249
1	0.000014102	-2.509335767	2.470152293
1	-0.000031949	-4.674514621	1.248132928
1	-0.000037990	-4.674520819	-1.248129851
6	0.000009955	3.744234677	-0.689192621
1	-0.000008287	2.509342835	2.470156393
1	-0.000021611	-0.000001875	2.484648262
1	0.000013793	4.674519942	1.248130061
1	-0.000012132	2.509335498	-2.470153369
1	0.000024737	4.674514230	-1.248133321

Table S24. Cartesian coordinates of the DPA resonance structure obtained at the final stage of route 1 (t_1) optimal-force procedure, at the TDA-M06-2X/6-31+G(d,p) level.

6	1.349946802	2.501108361	0.607093869
6	0.706121945	1.263701471	0.485836747
6	1.414468476	0.020345206	0.299700216
6	-0.726596494	1.233819900	0.491929022
6	-1.414213837	-0.020402111	0.298900899
6	-0.705990521	-1.263759201	0.485273995
6	0.726708555	-1.233883746	0.492341227
6	0.622400498	3.687636538	0.805841726
6	-1.430585877	2.430829846	0.668580790
6	-1.349912606	-2.501202427	0.605861272
6	1.430587786	-2.430859926	0.669552517
6	0.759845601	-3.653345464	0.844988812
6	-0.622490218	-3.687721787	0.805044101
1	-2.430902003	-2.549380453	0.538976268
1	2.515755448	-2.407751628	0.674204372
1	1.331335336	-4.563579281	0.994215944
1	-1.153779300	-4.627661787	0.914415181

6	-0.759962903	3.653283102	0.844654327
1	2.430990385	2.549314451	0.541033902
1	1.153631426	4.627544801	0.915745602
1	-2.515758297	2.407766705	0.672369288
1	-1.331560925	4.563515557	0.993468074
6	-2.820337774	-0.000360262	-0.166908394
6	-3.081610774	0.513651552	-1.450526375
6	-3.895796123	-0.480179890	0.596342714
1	-2.251678447	0.894853358	-2.044156347
1	-3.712911950	-0.852619730	1.602588688
6	-4.374584970	0.521822145	-1.966353820
6	-5.194272585	-0.460414125	0.083832192
1	-4.553850067	0.909048195	-2.966681437
1	-6.017934847	-0.828406419	0.692490428
6	-5.436811802	0.032468931	-1.199142176
1	-6.447633139	0.040053461	-1.597083966
6	2.820520251	0.000244599	-0.166285318
6	3.896180553	0.481000383	0.596186956
6	3.081399184	-0.514571399	-1.449735939
1	3.713697449	0.853972360	1.602331106
1	2.251307268	-0.896477106	-2.042769423
6	5.194423317	0.461431634	0.083057095
6	4.374105585	-0.522481542	-1.966198456
1	6.018250798	0.830137973	0.691125825
1	4.553011632	-0.910259349	-2.966414400
6	5.436534530	-0.032147381	-1.199773781
1	6.447160561	-0.039519734	-1.598204523

Table S25. Cartesian coordinates of the DPA resonance structure obtained at the final stage of the route 2 (t_2) optimal-force procedure, at the TDA-M06-2X/6-31+G(d,p) level.

6	1.352708448	2.498653441	0.611098529
6	0.706870512	1.262806531	0.485596073
6	1.413833174	0.019132707	0.297726195
6	-0.725869384	1.234706432	0.489333672
6	-1.413739689	-0.019054917	0.297498712
6	-0.706827196	-1.262726277	0.485380163
6	0.725912183	-1.234644567	0.489378543
6	0.626304925	3.686108706	0.809524809
6	-1.428677455	2.432701886	0.663536194
6	-1.352731660	-2.498567058	0.610705436
6	1.428655464	-2.432652165	0.663705943
6	0.756188043	-3.653868877	0.842815555
6	-0.626391215	-3.686036908	0.809223965
1	-2.434246121	-2.544326318	0.548648639
1	2.513915878	-2.411224682	0.663878862
1	1.326612438	-4.565040574	0.990304858
1	-1.158694277	-4.625015675	0.922526723
6	-0.756276423	3.653918990	0.842838723
1	2.434230025	2.544474726	0.549220065

1	1.158577364	4.625089092	0.922970426
1	-2.513939329	2.411241607	0.663452771
1	-1.326746573	4.565076645	0.990236100
6	-2.820257368	-0.000090195	-0.167889381
6	-3.079739279	0.494464635	-1.459810003
6	-3.896884680	-0.462747676	0.604946713
1	-2.248176655	0.862147284	-2.060641076
1	-3.713925972	-0.821383328	1.616654426
6	-4.373421578	0.500599125	-1.974154003
6	-5.196018563	-0.444308792	0.094004418
1	-4.551949791	0.872407974	-2.980872312
1	-6.020996625	-0.798192241	0.710024186
6	-5.437324815	0.029353718	-1.196950842
1	-6.448652168	0.035869697	-1.593599267
6	2.820321132	0.000112834	-0.167718035
6	3.896985926	0.463353885	0.604719393
6	3.079724537	-0.495141735	-1.459397226
1	3.714139484	0.822457623	1.616280193
1	2.248149209	-0.863281825	-2.059920792
6	5.196048475	0.444823178	0.093601562
6	4.373330567	-0.501366360	-1.973921557
1	6.021058136	0.799159572	0.709324825
1	4.551780809	-0.873708198	-2.980457001
6	5.437262599	-0.029511865	-1.197128743
1	6.448534812	-0.036091332	-1.593905583

7. Benchmarking against experimental 0-0 energies.

To assess the accuracy of the TDA-M06-2X/6-31+G(d,p) protocol, the adiabatic 0-0 energies (E_{00}) of the S_1 and T_1 states of BPEB, anthracene, and DPA were computed and compared with experimental band-origin values (Tables S26–S28). For each system, the S_0, S_1, and T_1 geometries were independently optimized and confirmed as true minima by analytical frequency calculations, and E_{00} was evaluated including zero-point energy corrections. In all cases the method reproduces the experimental band origins within ≈ 0.1–0.16 eV, supporting the reliability of the computed singlet–triplet energetics along the resonance routes.

7.1. Case BPEB

The experimental references for BPEB are band-origin (0-0) energies: S_1 = 3.86 eV from jet-cooled fluorescence-excitation spectroscopy in the gas phase [S2], and T_1 = 2.42 eV from the 77 K phosphorescence maximum in toluene, which closely approximates the T_1–S_0 0-0 energy [S3]. To compare on an equal footing, S_0, S_1 and T_1 were independently optimized with analytical frequencies (true minima). As shown in Table S26, the method reproduces the experimental band origins within its expected accuracy: S_1 and T_1 are each recovered to within ≈ 0.1 eV.

Table S26. Calculated zero–zero (0–0) transition energies (E_{00}) of BPEB compared with the available experimental reference values. All energies and deviations are reported in eV, with $\Delta = E_{00}^{\text{calc}} - E_{00}^{\text{exp}}$.

Quantity	Calc	Exp	Δ
$E_{00}(S_1)$	3.73	3.86[a]	−0.13
$E_{00}(T_1)$	2.51	2.42[b]	+0.09

[a] Gas-phase 0-0, jet-cooled.[2] [b] Phosphorescence maximum, 77 K in toluene (≈ 0-0).[S3].

7.2. Case Anthracene

The experimental references are also gas-phase band-origin (0-0) energies: T_1 = 1.872 eV from slow-electron velocity-map imaging (SEVI) photoelectron spectroscopy of the cold radical anion [S4], which gives the adiabatic S_0–T_1 splitting directly, and S_1 = 3.43 eV from the gas-phase fluorescence band origin [S5]. Following the same 0-0 protocol as before, S_0, S_1 and T_1 were independently optimized and their E_{00} energies evaluated. As shown in Table S27, both states are reproduced to within ≈ 0.14 eV, and the two errors share the same sign and therefore partly cancel in relative terms.

Table S27. Calculated zero–zero (0–0) transition energies (E_{00}) of Anthracene compared with the available experimental reference values. All energies and deviations are reported in eV, with $\Delta = E_{00}^{\text{calc}} - E_{00}^{\text{exp}}$.

Quantity	Calc	Exp	Δ
$E_{00}(S_1)$	3.57	3.43[a]	+0.14
$E_{00}(T_1)$	2.01	1.872[b]	+0.14

[a] $S_1 \leftarrow S_0$ band origin, gas-phase fluorescence [S5]. [b] S_0–T_1 adiabatic 0-0 splitting, SEVI [S4].

7.3. Case DPA

For DPA, both experimental references are again band-origin (0-0) energies, obtained by different techniques than before. T_1 (1.77 eV) is from nanosecond flash photolysis [S6], and S_1 (3.27 eV) is the 0-0 origin of the $S_1 \leftarrow S_0$ transition measured by fluorescence-excitation spectroscopy of jet-cooled DPA in the gas phase [S7]. Following the same 0-0 protocol as before, both states were evaluated as E_{00} energies. As

shown in Table S28, both are reproduced within the expected accuracy of the method, with deviations of +0.11 eV for S_1 and +0.16 eV for T_1.

Table S28. Calculated zero–zero (0–0) transition energies (E_{00}) of DPA compared with the available experimental reference values. All energies and deviations are reported in eV, with $\Delta = E_{00}^{\mathrm{calc}} - E_{00}^{\mathrm{exp}}$.

Quantity	Calc	Exp	Δ
$E_{00}(S_1)$	3.38	3.27[a]	+0.11
$E_{00}(T_1)$	1.93	1.77[b]	+0.16

[a] 0-0 origin, gas-phase fluorescence-excitation spectroscopy of jet-cooled DPA [S7]. [b] ns laser flash photolysis [S6]

8. References

[S1]. Lu, T. A Comprehensive Electron Wavefunction Analysis Toolbox for Chemists, Multiwfn. *The Journal of Chemical Physics* **2024**, *161* (8), 082503. https://doi.org/10.1063/5.0216272.

[S2]. Suzuki, R.; Chiba, K.; Tanaka, S.; Okuyama, K. Electronic Spectra of Jet-Cooled 1,4-Bis(Phenylethynyl)Benzene: Strength in π-Electron Conjugation and Two Large-Amplitude Torsional Motions. *J. Chem. Phys*. **2024**, *160* (2), 024301. https://doi.org/10.1063/5.0176162.

[S3]. Lardani, D.; Ronchi, A.; Hu, X.; Monguzzi, A.; Weder, C. Bis(Phenylethynyl)Benzenes Enable Stable Visible-to-Ultraviolet Sensitized Triplet–Triplet Annihilation Upconversion. *J. Mater. Chem. C* **2025**, *13* (36), 18796–18804. https://doi.org/10.1039/D5TC02434J.

[S4]. Kregel, S. J.; Thurston, G. K.; Garand, E. Photoelectron Spectroscopy of Anthracene and Fluoranthene Radical Anions. *J. Chem. Phys*. **2018**, *148* (23), 234306. https://doi.org/10.1063/1.5036757.

[S5]. Lambert, W. R.; Felker, P. M.; Syage, J. A.; Zewail, A. H. Jet Spectroscopy of Anthracene and Deuterated Anthracenes. *J. Chem. Phys*. **1984**, *81* (5), 2195–2208. https://doi.org/10.1063/1.447922.

[S6]. Chattopadhyay, S. K.; Kumar, C. V.; Das, P. K. Triplet-Related Photophysics of 9,10-Diphenylanthracene. A Kinetic Study of Reversible Energy Transfer from Anthracene Triplet by Nanosecond Laser Flash Photolysis. *Chem. Phys. Lett*. **1983**, *98* (3), 250–254. https://doi.org/10.1016/0009-2614(83)87160-7.

[S7]. Tanaka, F.; Hirayama, S.; Shobatake, K. Microscopic Solvent Effects on the S_1–T_2 Intersystem Crossing of 9,10-Diphenylanthracene in Supersonic Free Jets. *Chem. Phys. Lett*. **1989**, *164* (4), 335–340. https://doi.org/10.1016/0009-2614(89)85215-7.
